# IFN-**γ**–induced trained immunity enhances killing of priority pathogens in healthy and genetically vulnerable individuals

**DOI:** 10.1172/jci.insight.195866

**Published:** 2026-02-10

**Authors:** Dearbhla M. Murphy, Isabella Batten, Aoife O’Farrell, Simon R. Carlile, Sinead A. O’Rourke, Chloe Court, Brenda Morris, Gina Leisching, Gráinne Jameson, Sarah A. Connolly, Adam H. Dyer, John P. McGrath, Emma McNally, Olivia Sandby-Thomas, Anjali Yennemadi, Conor M. Finlay, Clíona Ní Cheallaigh, Jean Dunne, Cilian Ó Maoldomhnaigh, Laura E. Gleeson, Aisling Dunne, Nollaig Bourke, Reinout van Crevel, Donal J. Cox, Niall Conlon, Arjun Raj, Rachel M. McLoughlin, Joseph Keane, Sharee A. Basdeo

**Affiliations:** 1 School of Medicine, Trinity Translational Medicine Institute, St. James’s Hospital, Trinity College Dublin, University of Dublin, Dublin, Ireland.; 2 Department of Bioengineering, School of Engineering and Applied Sciences, University of Pennsylvania, Philadelphia, Pennsylvania, USA.; 3 School of Biochemistry and Immunology, Trinity Biomedical Sciences Institute, Trinity College Dublin, University of Dublin, Dublin, Ireland.; 4 National Tuberculosis Centre, Department of Respiratory Medicine, and; 5 Department of Clinical Immunology, St. James’s Hospital, Dublin, Ireland.; 6 Department of Internal Medicine and Radboud Community for Infectious Diseases, Radboud University Medical Centre, Nijmegen, Netherlands.; 7 Department of Genetics, Perelman School of Medicine, University of Pennsylvania, Philadelphia, Pennsylvania, USA.

**Keywords:** Immunology, Infectious disease, Bacterial infections, Macrophages, Preventative medicine

## Abstract

Infectious diseases remain a global health challenge, driven by increasing antimicrobial resistance and the threat of emerging epidemics. *Mycobacterium tuberculosis* and *Staphylococcus aureus* are leading causes of mortality worldwide. Trained immunity — a form of innate immune memory — offers a promising approach to enhance pathogen clearance. Here, we demonstrate that IFN-γ induces trained immunity in human monocytes through a mechanism involving mTORC1 activation, glutaminolysis, and epigenetic remodeling. Macrophages derived from IFN-γ–trained monocytes exhibited increased glycolytic activity with enhanced cytokine and chemokine responses upon stimulation or infection. Crucially, trained macrophages had increased production of reactive oxygen species, which mediated enhanced bactericidal activity against methicillin-resistant *S*. *aureus and M*. *tuberculosis*. Furthermore, ATAC-sequencing analysis of IFN-γ–trained macrophages revealed increased chromatin accessibility in regions associated with host defense. Last, IFN-γ training restored impaired innate responses in macrophages from individuals homozygous for the *TIRAP* 180L polymorphism, a genetic variant associated with increased susceptibility to infection. These findings establish IFN-γ as a potent inducer of trained immunity in human monocytes and support its potential as a host-directed strategy to strengthen antimicrobial defenses, particularly in genetically susceptible individuals and high-risk clinical contexts.

## Abstract 2

Immune cells that defend against early infection can be trained to better fight tuberculosis and MRSA, especially in vulnerable individuals.

## Graphical abstract

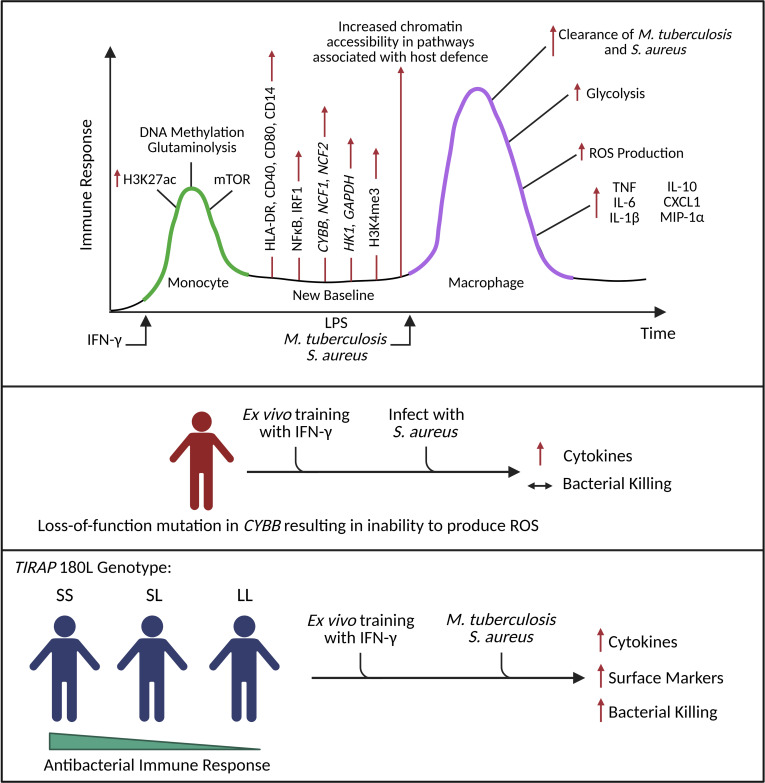

## Introduction

Trained immunity metabolically and epigenetically reprograms innate immune cells, altering their response to pathogens (1–7). Training murine myeloid cells in vivo enhances metabolism and cytokine production, resulting in improved pathogen clearance (1, 8–10). IFN-γ is critical for protection against multiple pathogens as it enhances antimicrobial immunity by driving autophagy, mediated through the interaction of the IFNGR and Mal (11, 12), iNOS (13, 14), and ROS production (13, 15). IFN-γ also activates glycolysis and oxidative phosphorylation in macrophages, aiding their proinflammatory function (13, 16–18). In vivo murine studies identified a critical role for IFN-γ, derived from natural killer or T cells, for the induction of trained immunity (8, 10, 19, 20); however, the impact of IFN-γ training on human myeloid cells is unexplored.

A knowledge gap exists surrounding the translation of trained immunity toward clinical benefit (21, 22). We sought to define the role of IFN-γ in training human monocytes and its effect on subsequent infection with *Mycobacterium tuberculosis* (M.tb) or methicillin-resistant *Staphylococcus* *aureus* (MRSA), which are both on the World Health Organization’s bacterial pathogens priority list (23). We also examined the benefits of trained immunity for individuals who are homozygous for the single-nucleotide polymorphism (SNP) 180L in their *TIRAP* gene. *TIRAP* 180L is associated with altered susceptibility to infections due to compromised TLR2, TLR4, and IFN-γ signaling impairing cytokine production (11, 24–26).

We demonstrate that IFN-γ trains human monocytes, which is mediated by mammalian target of rapamycin (mTOR) signaling, glutamine metabolism, and epigenetic imprinting. Macrophages derived from IFN-γ–trained monocytes (IFN-γ–trained macrophages) have an altered baseline phenotype and produce more cytokines and chemokines and enhance glycolysis upon stimulation, compared with untrained controls. IFN-γ–trained macrophages also produced more ROS, which was mechanistically associated with improved bacterial killing of M.tb or MRSA. Assay for transposase-accessible chromatin using sequencing (ATAC sequencing) corroborated that IFN-γ–trained macrophages had increased chromatin accessibility in regions associated with the host response to bacteria, ROS regulation, and regulation of nitric oxide biosynthesis. Finally, IFN-γ training enhanced cytokine responses and MRSA killing in macrophages from *TIRAP* 180L homozygotes, providing evidence that trained immunity has potential clinical benefit to individuals who are vulnerable to infection.

## Results

### Macrophages derived from IFN-γ–trained human monocytes had increased expression of activation markers.

**Figure 1:**
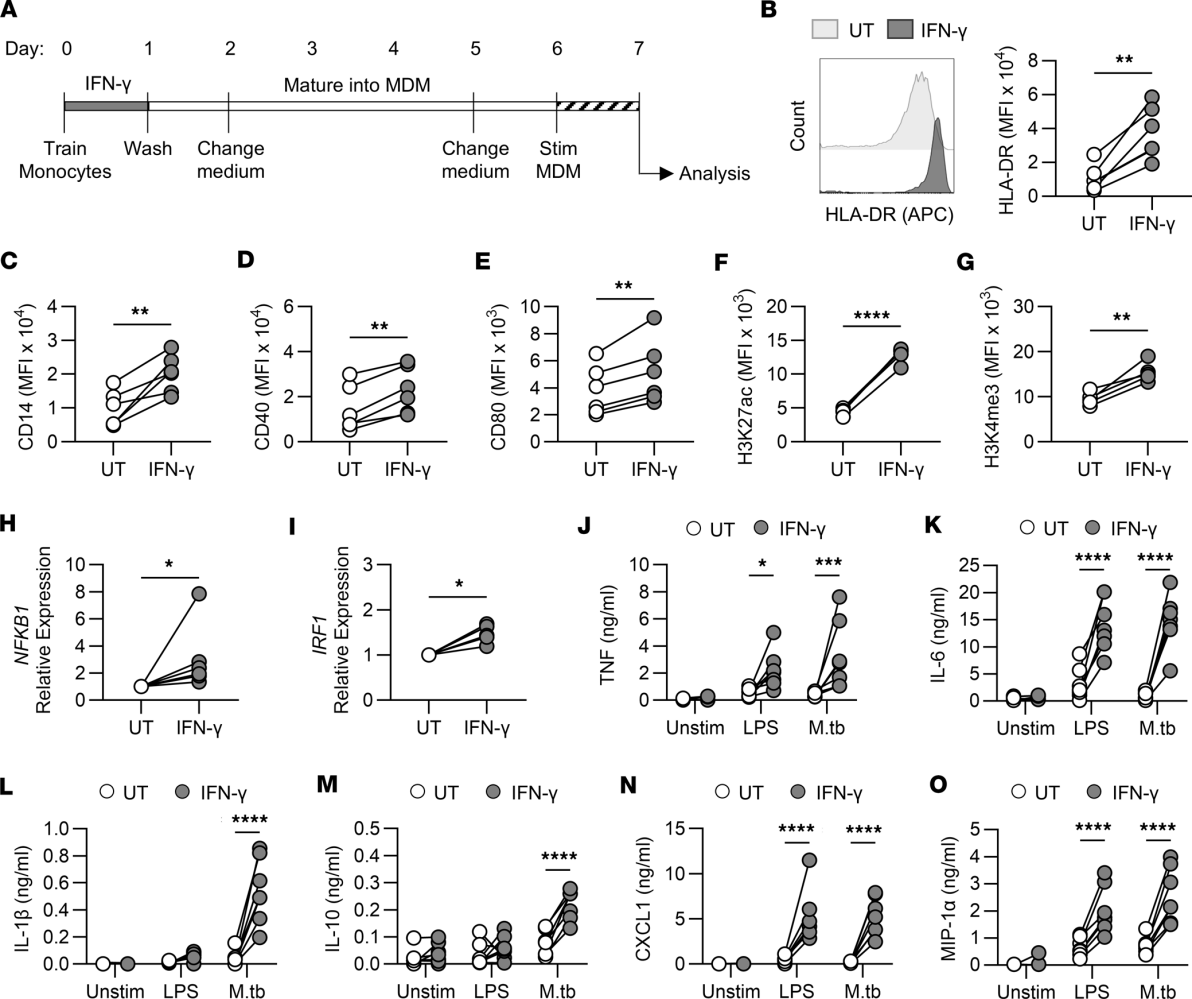
IFN-γ induces trained immunity in human monocytes. Enriched monocytes were left untrained (UT; white) or trained with IFN-γ (10 ng/mL; gray) for 24 hours. Cells were differentiated into MDM. (**A**) Schematic of training assay. (**B**–**G**) Expression of HLA-DR (**B**), CD14 (**C**), CD40 (**D**), CD80 (**E**), H3K27ac (**F**), or H3K4me3 (**G**) on unstimulated MDM on day 7 measured by flow cytometry. MFI, median fluorescence intensity. (**H** and **I**) Expression (relative to untrained) of *NFKB1* (**H**) or *IRF1* (**I**) in unstimulated MDM on day 7 measured by qPCR. (**J**–**O**) On day 6, MDM were stimulated with LPS (10 ng/mL) or irradiated M.tb (10 μg/mL) for 24 hours, and TNF (**J**), IL-6 (**K**), IL-1β (**L**), IL-10 (**M**), CXCL1 (**N**), or MIP-1α (**O**) was measured by ELISA. Each dot represents an individual donor, *n* = 6 (**B**–**E**, **H**, and **I**), *n* = 5 (**F** and **G**), or *n* = 7 (**J**–**O**), with paired data joined by a line. *P < 0.05, **P < 0.01, ***P < 0.001, ****P < 0.0001 determined using a paired *t* test (**B**–**I**) or a 2-way ANOVA with Šidák’s multiple-comparison test (**J**–**O**).

The training assay timeline is shown in Figure 1A. On day 7 of culture, monocyte-derived macrophages (MDM) were routinely greater than 95% pure (Supplemental Figure 1, A–C; supplemental material available online with this article; https://doi.org/10.1172/jci.insight.195866DS1). No significant differences in cell viability were observed (Supplemental Figure 2, A and B).

Activation marker expression in unstimulated IFN-γ–trained and untrained MDM was assessed on day 7 using flow cytometry. IFN-γ training increased expression of HLA-DR, CD14, CD40, and CD80 (Figure 1, B–E); did not enhance CD86 (Supplemental Figure 2C); and decreased CD83 expression (Supplemental Figure 2D). This phenotype is associated with trained immunity (1, 10, 27) and demonstrates that a single exposure of monocytes to IFN-γ can induce phenotypic changes that are maintained following MDM differentiation.

### IFN-γ training increased acetylation and methylation of histone 3.

As epigenetic reprogramming is essential for the induction of trained immunity (1, 2, 5, 6, 10, 28, 29), we sought to determine whether IFN-γ training induced epigenetic alterations in human monocytes and whether these were maintained after differentiation into MDM. H3K27 acetylation (H3K27ac) and H3K4 trimethylation (H3K4me3), commonly induced by training (1, 4, 6, 29), were measured using flow cytometry. After 24 hours, IFN-γ significantly increased H3K27ac (Supplemental Figure 2E) but not H3K4me3 (Supplemental Figure 2F) in monocytes. On day 7, IFN-γ training significantly enhanced H3K27ac (Figure 1F) and H3K4me3 (Figure 1G) in differentiated MDM. Collectively these data show that IFN-γ induces epigenetic reprogramming.

### Training of human monocytes with IFN-γ enhanced cytokine and chemokine production from MDM in response to bacterial stimuli.

To examine the functional effects of IFN-γ training, we quantified the relative mRNA expression of NF-κB and IRF1, known regulators of the cytokine response (30–33). IFN-γ training significantly enhanced MDM expression of *NFKB1* (Figure 1H) and *IRF1* (Figure 1I). When stimulated with LPS or M.tb, IFN-γ–trained MDM produced significantly higher concentrations of TNF (Figure 1J) and IL-6 (Figure 1K). IFN-γ–trained MDM produced more IL-1β (Figure 1L) and IL-10 (Figure 1M) in response to M.tb, but not LPS. As LPS signals through TLR4 (34) and M.tb signals through both TLR2 and TLR4 (35), we examined the effect of IFN-γ training on TLR-2 and TLR-4 expression but found no changes (Supplemental Figure 2, G and H). IFN-γ training significantly increased production of the chemokines CXCL1 and MIP-1α (Figure 1, N and O) following LPS or M.tb stimulation. No differences in viability were observed (Supplemental Figure 2I). Collectively, these findings show that training monocytes with IFN-γ enhances cytokine and chemokine production from MDM stimulated with either LPS or M.tb.

IFN-γ can drive subsequent IFN-γ production, through TBET activation (36). However, IFN-γ training did not significantly alter *TBET* expression or enhance IFN-γ production following stimulation (Supplemental Figure 2, J and K).

Another T cell cytokine, IL-17, failed to induce trained immunity in vitro (Supplemental Figure 2L). Furthermore, IFN-γ–trained MDM had enhanced production of IL-6 and IL-1β in response to M.tb stimulation up to day 28 after training (Supplemental Figure 2, N and O), suggesting that IFN-γ training can be maintained for up to a month in vitro.

### IFN-γ training activated mTOR in human monocytes and enhanced glycolytic gene expression in MDM.

**Figure 2:**
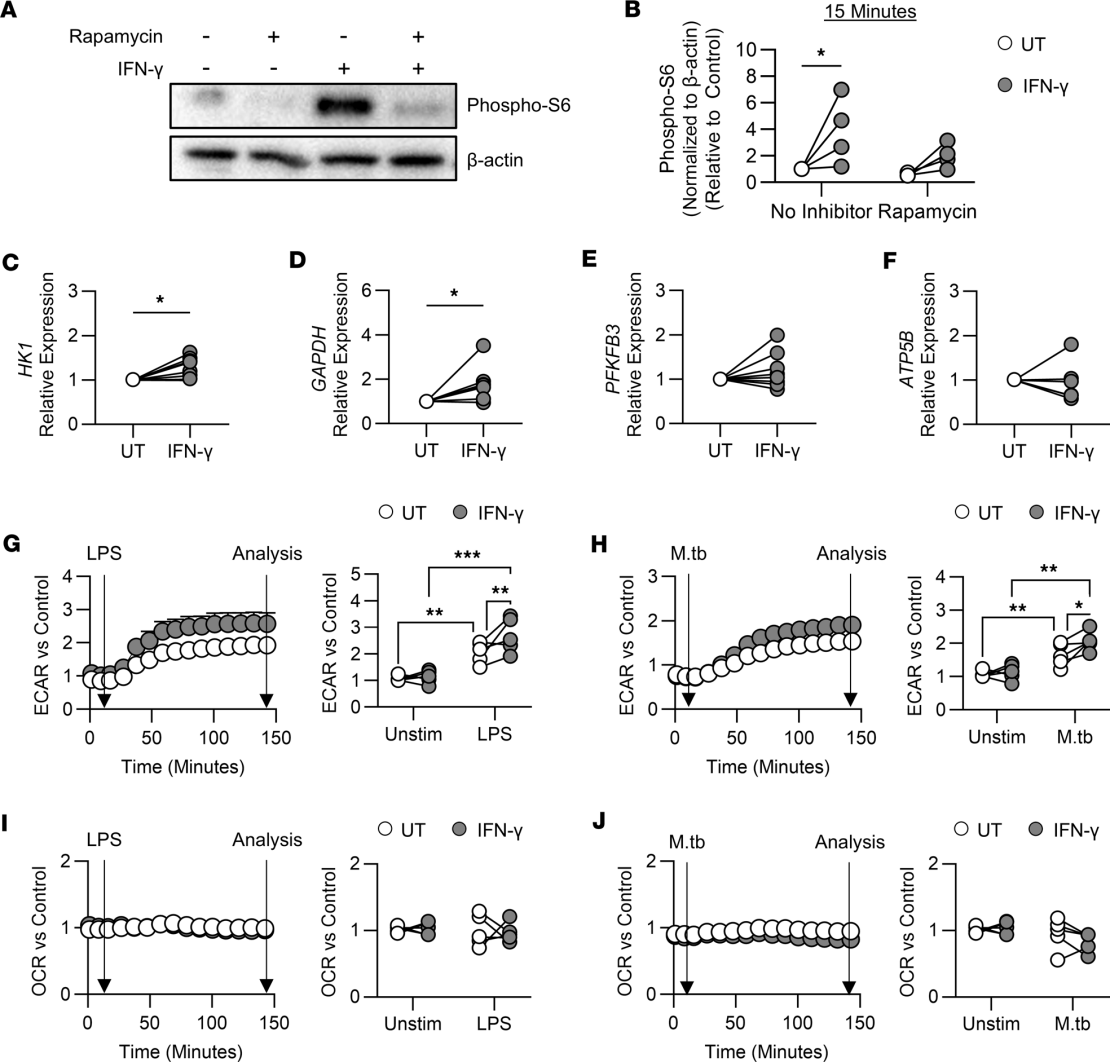
IFN-γ training metabolically reprograms human macrophages. (**A** and **B**) Sorted monocytes were left untreated or treated with IFN-γ (10 ng/mL; gray) for 15 minutes with or without rapamycin (50 nM). (**A**) Representative Western blot of phospho-S6 and β-actin density for each sample. (**B**) Phospho-S6 density normalized to β-actin, relative to untreated untrained control, measured by densitometry. (**C**–**J**) Enriched monocytes were left untrained (UT; white) or trained with IFN-γ (10 ng/mL; gray) for 24 hours. Cells were differentiated into MDM. (**C**–**F**) Relative expression (compared with untrained) of HK1 (**C**), GAPDH (**D**), PFKFB3 (**E**), or ATP5B (**F**) in unstimulated MDM on day 7 measured by qPCR. (**G**–**J**) On day 6, MDM metabolism was assessed using the Seahorse XFe24 analyzer. The arrows depict the time of stimulation and data analysis. (**G** and **H**) Relative (to untrained unstimulated) ECAR in response to LPS (10 ng/mL) (**G**) or irradiated M.tb (10 μg/mL) (**H**). (**I** and **J**) Relative (to untrained unstimulated) OCR in response to LPS (10 ng/mL) (**I**) or irradiated M.tb (10 μg/mL) (**J**). Each dot represents an individual donor, *n* = 4 (**A** and **B**), *n* = 6–8 (**C**–**F**), or *n* = 5 (**G**–**J**), with paired data joined by a line. *P < 0.05, **P < 0.01, ***P < 0.001 determined using a 2-way ANOVA with Fisher’s least significant difference (LSD) test (**B** and **G**–**J**) or a paired *t* test (**C**–**F**).

Metabolic reprogramming is critical for inducing trained immunity (3–5). mTOR regulates glycolysis and is crucial for trained immunity induced by β-glucan or Bacille Calmette-Guérin (BCG) (3–5). Thus, we determined whether IFN-γ activated mTOR complex 1 (mTORC1) in monocytes by measuring the phosphorylation of S6, a marker of mTORC1 activation (37, 38). IFN-γ enhanced phospho-S6 (Figure 2, A and B), which was inhibited by rapamycin (Figure 2B), suggesting mTORC1-dependent activation. Since mTOR regulates metabolic gene transcription, we quantified the gene expression of glycolytic enzymes and ATP synthase, an enzyme intrinsic to ATP production during oxidative phosphorylation. IFN-γ training significantly increased expression of hexokinase 1 (*HK1*; Figure 2C) and glyceraldehyde 3-phosphate dehydrogenase (*GAPDH*; Figure 2D). IFN-γ training did not alter the expression of other glycolytic enzymes: glucose-6-phosphate isomerase (*GPI*; Supplemental Figure 3A), phosphofructokinase (*PFKFB3*; Figure 2E), or pyruvate kinase (*PKM2*; Supplemental Figure 3B). Furthermore, IFN-γ training did not affect ATP synthase (*ATP5B*; Figure 2F). These data suggest that training monocytes with IFN-γ reprograms gene expression of MDM metabolism toward a glycolytic state.

### IFN-γ training significantly enhanced the glycolytic response to bacterial stimuli.

Enhanced glycolytic metabolism is a hallmark of trained immunity and essential for mediating antimicrobial responses in human macrophages (3, 5, 7, 18, 39). To assess changes in MDM metabolism in response to LPS or M.tb stimulation, we performed analyses on IFN-γ–trained and untrained MDM using the Seahorse XFe24 analyzer, which measures the extracellular acidification and oxygen consumption rates (ECAR and OCR, respectively).

At baseline, IFN-γ training did not enhance ECAR (Figure 2, G and H). When stimulated with LPS or M.tb, IFN-γ–trained MDM had a significantly higher ECAR compared with untrained MDM (Figure 2, G and H). There was no significant difference in the OCR of IFN-γ–trained MDM compared with untrained MDM (Figure 2, I and J). In keeping with this, the ECAR/OCR ratio (39, 40) — calculated by division of the change in ECAR by the change in OCR — was significantly higher in IFN-γ–trained MDM in response to LPS or M.tb (Supplemental Figure 3C), which suggests that IFN-γ training preferentially enhances flux through glycolysis in response to stimulation.

### IFN-γ–induced trained immunity required mTOR activation, glutaminolysis, and epigenetic modifications.

**Figure 3:**
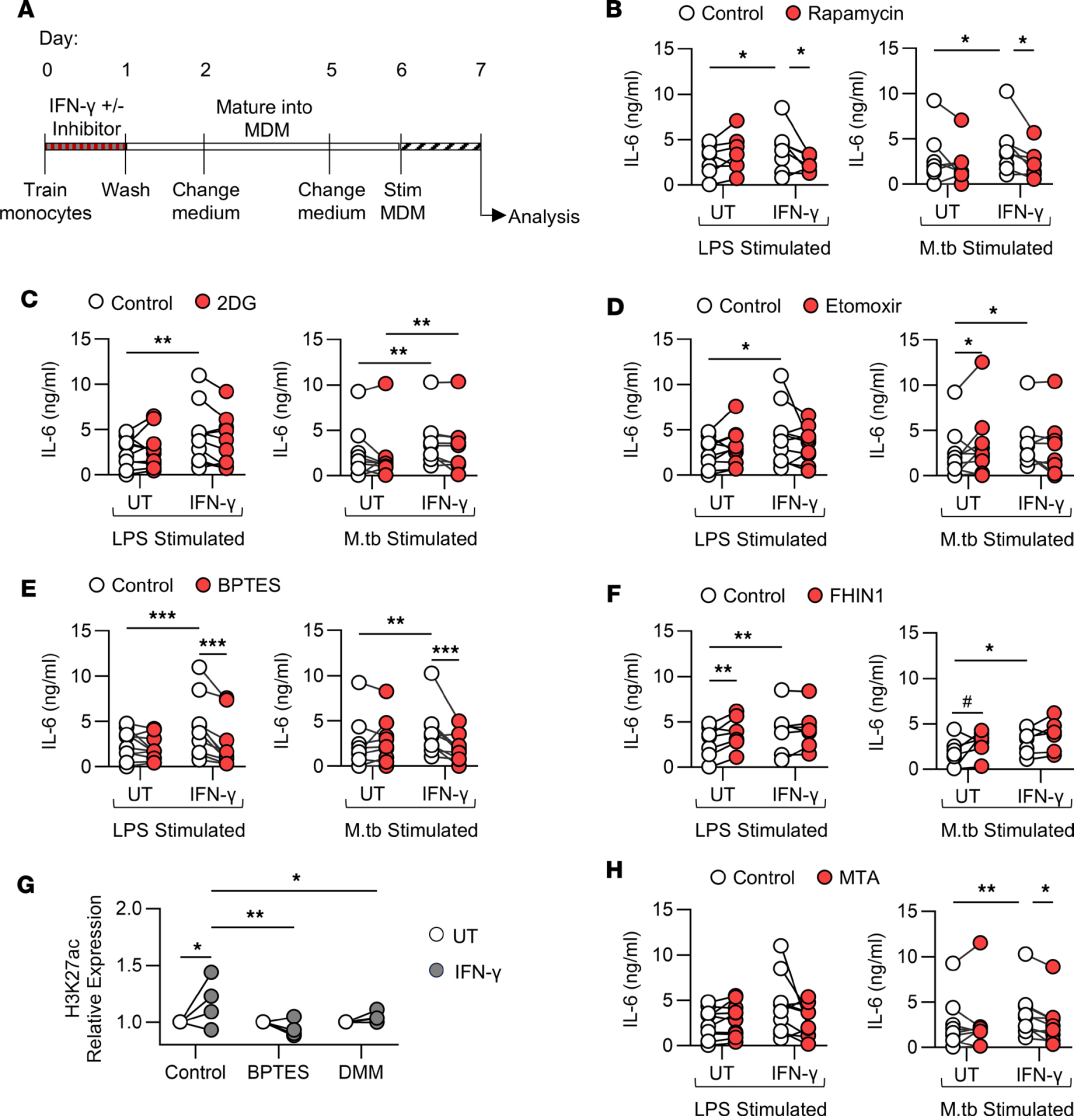
IFN-γ–induced trained immunity requires mTOR activation, glutaminolysis, and epigenetic modifications. (**A**–**H**) Enriched monocytes were left untrained or trained with IFN-γ (10 ng/mL) alone (white) or in the presence of the inhibitor (red) rapamycin (50 nM) (**B**), 2DG (1 mM) (**C**), etomoxir (5 μM) (**D**), BPTES (50 μM) (**E**), FHIN1 (10 μM) (**F**), BPTES (50 μM) or DMM (1 mM) (**G**), or MTA (1 mM) (**H**) for 24 hours. Cells were then differentiated into MDM. (**B**–**F** and **H**) On day 6, MDM were stimulated with LPS (10 ng/mL; left) or irradiated M.tb (10 μg/mL; right) for 24 hours, and IL-6 was measured by ELISA. (**G**) On day 7, H3K27ac was measured in unstimulated MDM using flow cytometry. Each dot represents an individual donor, *n* = 7–8 (**B**), *n* = 9–10 (**C**–**E** and **H**), *n* = 7 (**F**), or *n* = 4 (**G**), with paired data joined by a line. *P < 0.05, **P < 0.01, ***P < 0.001 determined using a mixed-model 2-way ANOVA with Fisher’s LSD test. (**F**) Data were analyzed by 2-way ANOVA; however, results were not statistically significant. ^#^*P* < 0.05, paired *t* test comparing untrained MDM with untrained MDM treated with FHIN in response to M.tb stimulation.

Having established that MDM differentiated from IFN-γ–trained monocytes have altered metabolism and epigenetics, we sought to determine whether these adaptations are critical for IFN-γ training at the time of exposure. A timeline of the training assay with inhibitors is shown (Figure 3A). Increased production of the proinflammatory cytokine IL-6 following training is well established (4, 5, 9, 41) and was measured following stimulation to assess successful induction of training.

Having shown that mTOR is significantly more active in monocytes following IFN-γ exposure (Figure 2, A and B), the effect of rapamycin was determined. Rapamycin did not alter IL-6 production in untrained MDM in response to LPS or M.tb (Figure 3B). IFN-γ–trained MDM produced more IL-6 in response to LPS or M.tb compared with untrained MDM; however, when monocytes were trained in the presence of rapamycin, this enhanced production was abrogated (Figure 3B), suggesting that rapamycin inhibited the induction of training.

Glycolysis is critical for inducing training with BCG, β-glucan, and oxidized low-density lipoproteins (oxLDLs) (3, 5, 7). Our data indicate that IFN-γ–trained MDM have enhanced expression of glycolytic enzymes and preferentially use glycolysis upon stimulation (Figure 2, C, D, G, and H, and Supplemental Figure 3C). Inhibition of glycolysis with 2-deoxyglucose (2DG) during monocyte training did not alter IL-6 production from IFN-γ–trained MDM in response to LPS (Figure 3C). Furthermore, IFN-γ–trained MDM produced more IL-6 in response to M.tb compared with untrained MDM in the presence or absence of 2DG (Figure 3C). These findings suggest that glycolysis is not essential for the induction of trained immunity by IFN-γ.

Fatty acid oxidation (FAO) is important for the induction of trained immunity by β-glucan and oxLDLs (42, 43). When stimulated with LPS, IFN-γ–trained MDM produced more IL-6 compared with untrained MDM (Figure 3D), and etomoxir treatment (which inhibits FAO) had no significant effect on IL-6 production from untrained or IFN-γ–trained MDM (Figure 3D). Interestingly, treating untrained monocytes with etomoxir enhanced IL-6 production from untrained MDM in response to M.tb (Figure 3D). However, etomoxir had no significant impact on IL-6 production from IFN-γ–trained MDM in response to M.tb (Figure 3D). These findings suggest that FAO is not required for the induction of trained immunity by IFN-γ but indicate that fatty acids may have a role in the induction of trained immunity.

Glutaminolysis has been shown to be essential for the induction of trained immunity by β-glucan or BCG in human monocytes and by LPS in murine alveolar macrophages (4, 5, 41). Enhanced glutaminolysis leads to increased α-ketoglutarate production, which is fed into the tricarboxylic acid (TCA) cycle. α-ketoglutarate is converted to succinate, and then to fumarate, which drives the epigenetic changes required for trained immunity (4). Treating monocytes with bis-2-(5-phenylacetamido-1,2,4-thiadiazol-2-yl)ethyl sulfide (BPTES), which inhibits glutaminolysis, had no effect on IL-6 production from untrained MDM in response to LPS or M.tb (Figure 3E). However, BPTES reduced IL-6 production from IFN-γ–trained MDM in response to LPS or M.tb (Figure 3E), resulting in concentrations similar to those from untrained MDM. These data suggest a critical role for glutaminolysis in the induction of trained immunity by IFN-γ.

Fumarate is capable of inducing trained immunity in vitro and is essential for the induction of trained immunity by β-glucan (4). We used fumarate hydratase inhibitor 1 (FHIN1), which prevents conversion of fumarate to malate, leading to fumarate accumulation, which we postulated would recapitulate the effects of IFN-γ training in untrained cells. When treated with FHIN1, untrained MDM produced significantly higher concentrations of IL-6 in response to LPS or M.tb (Figure 3F); however, FHIN1 had no effect on IL-6 production from IFN-γ–trained MDM in response to LPS or M.tb (Figure 3F). These data suggest that fumarate may already be accumulating in IFN-γ–trained cells and therefore cannot be further enhanced by FHIN1. As fumarate accumulation downstream of glutaminolysis is important for inducing epigenetic changes in β-glucan–trained cells (4), we assessed the effects of inhibiting glutaminolysis and succinate dehydrogenase, the enzyme that converts succinate to fumarate, on H3K27ac, which was increased in monocytes 24 hours after IFN-γ (Supplemental Figure 2E). BPTES and dimethyl malonate (DMM) were used to inhibit glutaminolysis and succinate dehydrogenase, respectively. IFN-γ training enhanced H3K27ac in MDM (Figure 1F and Figure 3G); however, treatment with BPTES or DMM abrogated H3K27ac (Figure 3G). Taken together, these data show that glutaminolysis is essential for the induction of trained immunity by IFN-γ and suggest a role for fumarate in driving the epigenetic changes observed.

Finally, we evaluated DNA methylation in IFN-γ–induced trained immunity, since it has been demonstrated to be essential for inducing trained immunity with other training agents (1, 4–6). As H3K4me3 expression was not increased 24 hours after IFN-γ training (Supplemental Figure 2F), we used 5′-methylthioadenosine (MTA), which inhibits DNA methylation, to determine whether other methylation marks are necessary for inducing IFN-γ training. MTA did not significantly affect IL-6 production from LPS-stimulated cells (Figure 3H). When stimulated with M.tb, IFN-γ–trained MDM produced significantly higher concentrations of IL-6, and MTA treatment significantly decreased this IL-6 production (Figure 3H). These data suggest that DNA methylation is important for IFN-γ training in monocytes.

Collectively, these findings suggest that trained immunity induced by IFN-γ is mediated by activation of mTORC1, glutaminolysis, and DNA methylation in human monocytes. Inhibition of glutaminolysis or succinate dehydrogenase abrogated H3K27ac induced by IFN-γ, implicating glutamine metabolism and fumarate accumulation in driving the epigenetic changes required for IFN-γ–induced trained immunity.

### IFN-γ–trained MDM had enhanced capacity to kill M. tuberculosis.

**Figure 4:**
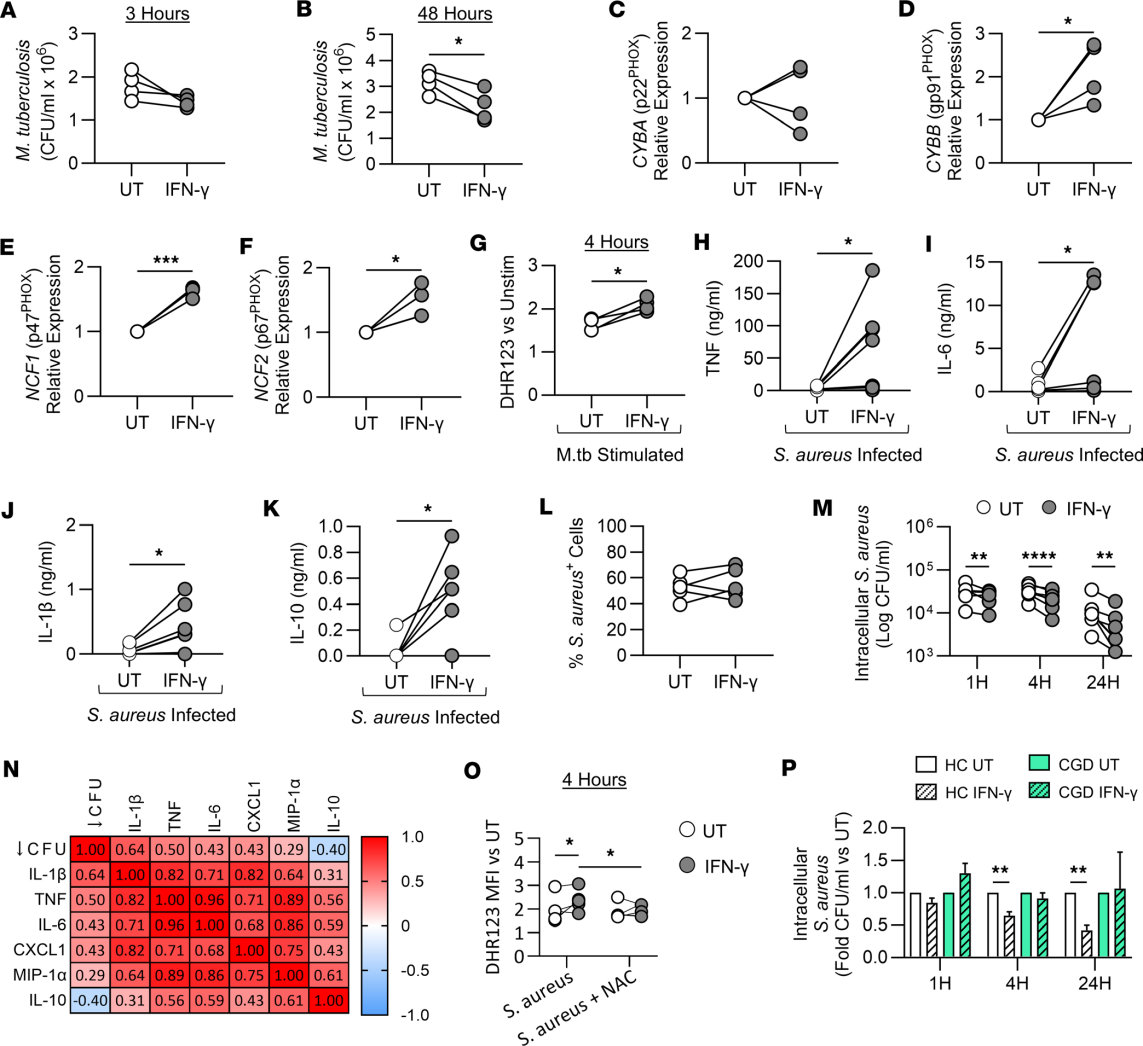
IFN-γ training increases the capacity of MDM to kill *S*. *aureus* and *M*. *tuberculosis* by enhancing ROS production. Enriched monocytes were left untrained (UT; white) or trained with IFN-γ (10 ng/mL; gray) for 24 hours. Cells were differentiated into MDM. On day 7, MDM were infected with *M*. *tuberculosis* (H37Rv; MOI 1–5) (**A** and **B**), stimulated with irradiated M.tb (10 μg/mL) (**G**), or infected with *S*. *aureus* (USA300; MOI 100:1) (**H**–**P**) for the indicated times. (**A** and **B**) Bacterial *M*. *tuberculosis* burden within MDM 3 and 48 hours after infection. (**C**–**F**) Relative expression (to untrained) of *CYBA* (**C**), *CYBB* (**D**), *NCF1* (**E**), or *NCF2* (**F**) in uninfected MDM on day 7 (qPCR). (**G**) MDM were stimulated with irradiated M.tb for 4 hours, and ROS (DHR123; relative to unstimulated MDM) was measured (flow cytometry). (**H**–**K**) TNF (**H**), IL-6 (**I**), IL-1β (**J**), or IL-10 (**K**) (ELISA) 24 hours after *S*. *aureus* infection. (**L**) MDM infected with CFSE-labeled *S*. *aureus* (%). (**M**) Intracellular bacterial *S*. *aureus* burden within MDM 1, 4, or 24 hours after gentamicin. (**N**) Spearman’s *r* correlation matrix: correlation of reduced CFU with IL-1β, TNF, IL-6, IL-10, CXCL1, or MIP-1α production. (**O**) MDM were infected with *S*. *aureus* alone or in the presence of NAC (10 mM) for 4 hours, and ROS was measured (flow cytometry). (**P**) Fold change in CFU/mL of untrained or IFN-γ–trained (stripes) MDM infected with *S*. *aureus* 1, 4, or 24 hours after gentamicin in healthy controls (HC; white) versus MDM from a patient with chronic granulomatous disease (CGD; green). Each dot represents an individual donor, *n* = 4 (**A**–**G**), *n* = 7 (**H**–**K**, **M**, and **N**), *n* = 5 (**L**), *n* = 6 (**O**), or HC *n* = 7, CGD *n* = 1 (**P**). Data are graphed as paired data joined by a line or the mean value ± SD. *P < 0.05, **P < 0.01, ***P < 0.001, ****P < 0.0001, paired *t* test (**A**–**L**) or 2-way ANOVA with Šidák’s multiple-comparison test (**M**), uncorrected Fisher’s LSD test (**O**), or Tukey’s multiple-comparison test (**P**).

Having established that IFN-γ can induce trained immunity in human monocytes, resulting in enhanced responsiveness of MDM to stimulation with irradiated M.tb, we sought to determine the impact of IFN-γ training on mycobacterial killing. Monocytes were left untrained or were trained with IFN-γ for 24 hours. On day 7, MDM were infected with live *M*. *tuberculosis* (H37Rv; MOI 1–5) for 3 hours, then washed to remove remaining extracellular bacteria and lysed (Figure 4A) or incubated for a further 48 hours (Figure 4B). Fluorescent latex beads were used to ascertain whether IFN-γ training affects MDM phagocytosis; however, no difference in phagocytosis was observed (Supplemental Figure 4A).

At 3 hours after infection, there was no difference in CFU/mL between trained and untrained MDM (Figure 4A). However, IFN-γ–trained MDM had significantly decreased CFU/mL of *M*. *tuberculosis* at 48 hours after infection (Figure 4B), indicative of enhanced bacterial killing.

### IFN-γ–trained MDM had enhanced gene expression of NADPH oxidase.

Since ROS production plays an integral role in killing intracellular bacteria such as M.tb (44) and drives IL-1β production (45, 46), we hypothesized that IFN-γ training may increase ROS production, resulting in the enhanced bactericidal effects observed.

The gene expression of 4 different subunits of the NADPH oxidase system in unstimulated untrained or IFN-γ–trained MDM was assessed by quantitative reverse transcription PCR. There was no difference in the expression of *CYBA* (p22^PHOX^; Figure 4C); however, IFN-γ–trained MDM had significantly increased expression of *CYBB* (gp91^PHOX^; Figure 4D), *NCF1* (p47^PHOX^; Figure 4E), and *NCF2* (p67^PHOX^; Figure 4F), suggesting that IFN-γ training may result in enhanced ROS production.

Next, we quantified total ROS production using dihydrorhodamine-123 (DHR123). Unstimulated IFN-γ–trained MDM produced significantly less intracellular ROS compared with untrained MDM (Supplemental Figure 4B), so ROS production was calculated relative to unstimulated MDM. Following stimulation with M.tb, IFN-γ–trained MDM exhibited significantly increased ROS production compared with untrained MDM (Figure 4G). Furthermore, IFN-γ–trained MDM produced significantly more mitochondrial ROS in response to M.tb compared with untrained MDM (Supplemental Figure 4C).

### IFN-γ training increased the capacity of MDM to kill MRSA, which correlated with enhanced cytokine production.

Since a key feature of trained immunity is that it is nonspecific, we examined the efficacy of IFN-γ–induced trained immunity against MRSA infection. When infected with MRSA, IFN-γ–trained MDM produced significantly higher concentrations of TNF, IL-6, IL-1β, IL-10 (Figure 4, H–K), CXCL1, and MIP-1α (Supplemental Figure 4, D and E) compared with untrained MDM. IFN-γ training had no effect on phagocytosis of CFSE-labeled MRSA by MDM at 1 hour after infection. Subsequently, CFU were enumerated at specific time points after gentamicin treatment to quantify intracellular bacterial loads within MDM. IFN-γ–trained MDM had significantly reduced intracellular bacterial load compared with untrained MDM at 1, 4, and 24 hours after gentamicin treatment (Figure 4M), indicative of enhanced intracellular bacterial killing.

Correlation of enhanced cytokine production with reduced MRSA CFU was assessed by a correlation matrix. CFU data were converted into a relative decrease in CFU at 24 hours (↓ CFU) in IFN-γ–trained MDM compared with untrained MDM. All cytokine data were converted into fold change in production from IFN-γ–trained cells compared with untrained cells in response to infection with MRSA. Spearman’s correlation shows a moderate positive correlation between the decrease in intracellular bacterial burden and the fold increase in the concentration of IL-1β (*r* = 0.64), TNF (*r* = 0.50), IL-6 (*r* = 0.43), CXCL1 (*r* = 0.43), and MIP-1α (*r* = 0.29; Figure 4N) at 24 hours after infection in IFN-γ–trained MDM. A negative correlation existed between decreased CFU and IL-10 production (*r* = –0.40; Figure 4N). An extended matrix was also plotted (Supplemental Figure 5).

### Enhanced ROS production in IFN-γ–trained MDM reduced MRSA bacterial loads.

Since the enhanced bactericidal capacity was evident at 1 and 4 hours, we postulated that ROS production may mediate this early antimicrobial effect. We quantified intracellular ROS production at 4 hours after infection with MRSA; IFN-γ–trained MDM produced significantly more ROS compared with untrained MDM (Figure 4O).

To determine whether enhanced ROS production mechanistically mediated enhanced bacterial clearance of MRSA in IFN-γ–trained MDM, cells were infected in the presence of the ROS inhibitor *N*-acetyl-l-cysteine (NAC; 10 mM) or vehicle control. In the presence of NAC, IFN-γ–trained MDM produced less ROS (Figure 4O and Supplemental Figure 6A), and the enhanced MRSA killing in IFN-γ–trained MDM was abrogated (Supplemental Figure 6B). To support the evidence indicating that ROS mechanistically mediated the bactericidal effects, monocytes with a mutation in the *CYBB* gene (gp91^PHOX^), which results in an inability to produce ROS, were isolated from a patient with chronic granulomatous disease (CGD). Since the *CYBB* gene was significantly upregulated with IFN-γ training (Figure 4D), and since patients with CGD have significant susceptibility to *S*. *aureus* infections (47, 48), we hypothesized that IFN-γ training would have no effect on bacterial killing in MDM that cannot produce ROS. IFN-γ–trained MDM with a loss-of-function (LOF) mutation in the *CYBB* gene produced higher concentrations of IL-6 and TNF (Supplemental Figure 6, C and D) and lower concentrations of IL-10 (Supplemental Figure 6E) compared with the untrained control but did not produce any IL-1β in response to MRSA infection (Supplemental Figure 6F). These data indicate that IFN-γ successfully induced trained immunity in monocytes from a patient with CGD, similar to healthy controls.

Next, MDM with a LOF mutation in the *CYBB* gene were infected with MRSA. There was no difference in the CFU/mL between untrained and IFN-γ–trained MDM from the CDG patient at 1, 4, and 24 hours (Supplemental Figure 6G). The fold change in CFU/mL of IFN-γ–trained MDM compared with untrained MDM was plotted for the healthy control donor (HC) MDM and the MDM with a LOF mutation in the *CYBB* gene (Figure 4P). IFN-γ–trained MDM from HCs had significantly decreased intracellular burden compared with untrained MDM at 4 and 24 hours after infection; however, IFN-γ–trained MDM with a LOF mutation in the *CYBB* gene did not exhibit any enhanced capacity to kill MRSA compared with their own untrained control MDM (Figure 4P).

Taken together these data indicate that IFN-γ–trained MDM produce significantly more ROS during infection, which contributes mechanistically to enhanced bacterial killing in comparison with untrained MDM.

### ATAC-sequencing of IFN-γ–trained MDM revealed increased chromatin accessibility in pathways associated with host defense.

As epigenetic modifications are essential for the induction, maintenance, and effects of trained immunity, we next assessed whether epigenetic modifications in IFN-γ–trained MDM were evident in regions associated with the functional effects observed. To this end, we repurposed a dataset from O’Farrell et al. (49) where they performed ATAC-sequencing of IFN-γ–trained MDM in 3 human donors.

**Figure 5:**
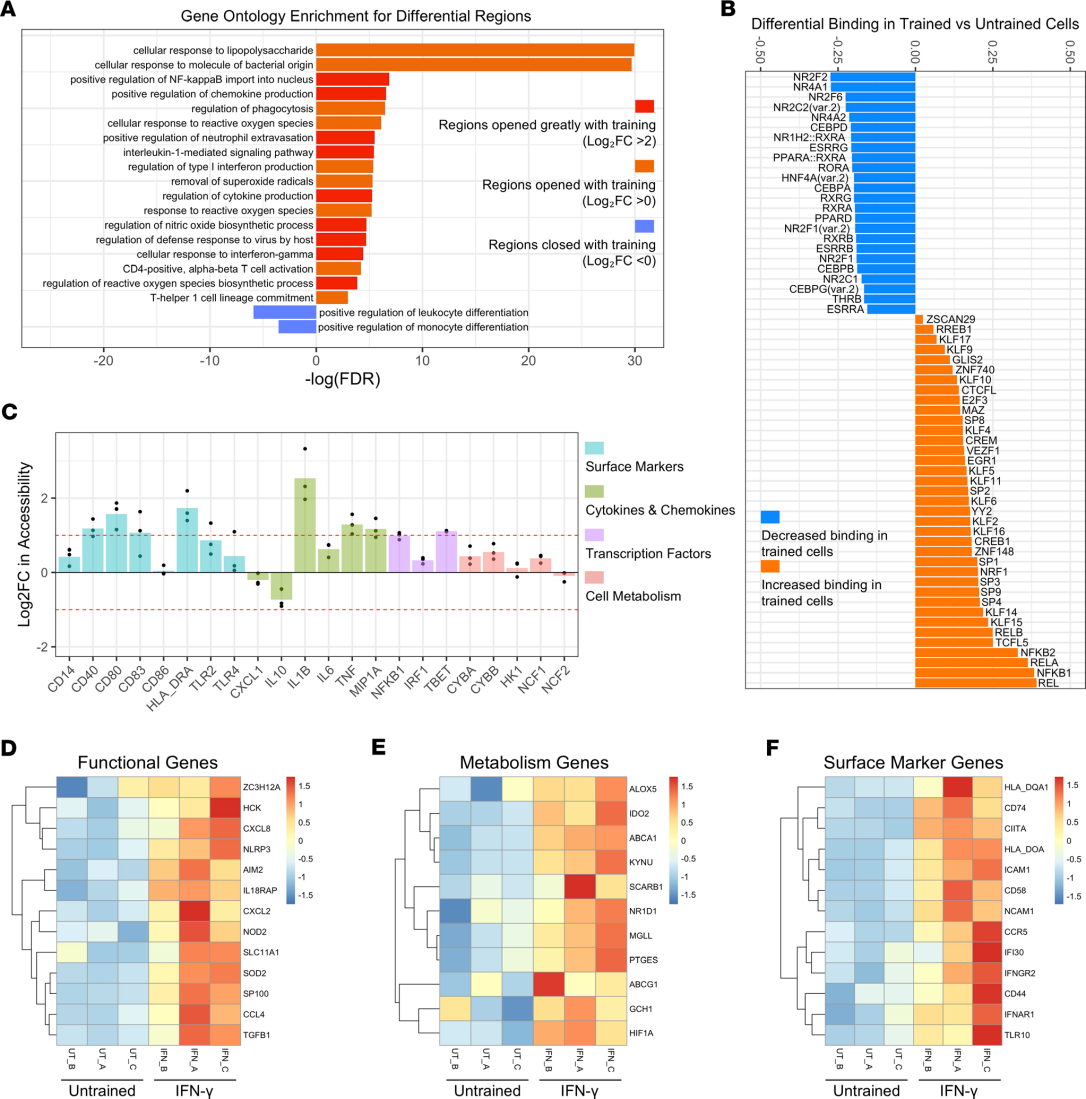
IFN-γ training epigenetically alters MDM. Sorted monocytes (*n* = 3 donors) were left untrained (UT) or trained with IFN-γ (50 ng/mL) for 24 hours. Cells were differentiated into MDM. On day 6, ATAC-sequencing was performed to determine epigenetic differences between UT and IFN-γ–trained MDM. (**A**) Gene ontology analysis showing selected enriched cellular pathways for regions that greatly increase in accessibility (red; log_2_FC > 2), regions that increase in accessibility (orange; log_2_FC > 0), and regions that decrease in accessibility (blue; log_2_FC < 0) in IFN-γ–trained cells. (**B**) TOBIAS plot showing the increase (orange) or decrease (blue) in transcription factor binding in IFN-γ–trained cells compared with UT cells. (**C**) Graph showing the log_2_FC in accessibility of candidate genes in IFN-γ–trained cells compared with UT cells. The genes are grouped as surface marker genes (blue), cytokine and chemokine genes (green), transcription factor genes (purple), and genes associated with cell metabolism (pink). Each point reflects one donor. (**D**–**F**) Heatmaps showing row-normalized accessibility at statistically significantly differential peak regions within candidate genes in IFN-γ–trained cells compared with UT cells grouped by functional genes (**D**), metabolism genes (**E**), and genes associated with cell surface marker expression (**F**). Graphs in **A**–**C** show log_2_ fold changes in IFN-γ–trained cells compared with UT cells. Graphs in **D**–**F** show overall accessibility at peaks of interest in both UT and IFN-γ–trained cells (see Methods).

We performed gene ontology analysis on altered regions (opened and closed) of the genome to assess which cellular pathways were impacted (Figure 5A). There was increased chromatin accessibility in pathways associated with cellular responses to bacteria and viruses, cytokine and chemokine production, regulation of phagocytosis and ROS production, and nitric oxide biosynthesis (Figure 5A), accompanied by decreased accessibility in pathways associated with monocyte differentiation (Figure 5A). Differential binding of transcription factors in IFN-γ–trained versus untrained cells showed decreased binding of *NR2F1*, *NR2F2*, *CEBPD*, and *CEBPB* and increased binding of *REL*, *NFKB1*, *NFKB2*, and *NRF1* (Figure 5B). Next, we examined whether the increased cell surface marker expression, cytokine and chemokine production, transcription factor expression, and cell metabolism examined in the IFN-γ–trained cells (Figures 1–4) were due to epigenetic alterations in the genes associated with these changes (Figure 5C). IFN-γ–trained MDM had increased chromatin accessibility in *CD40*, *CD80*, *CD83*, *HLADRA*, *IL1B*, *TNF*, and *MIP1A* (Figure 5C). Lastly, we examined the change in accessibility of genes associated with cell function (Figure 5D), metabolism (Figure 5E), and phenotype (Figure 5F). IFN-γ–trained MDM had increased accessibility at many genes associated with cell signaling and cytokine production (Figure 5D), genes associated with lipid and cholesterol metabolism (Figure 5E), and genes associated with antigen presentation, interferon signaling, and cell migration (Figure 5F). These findings indicate that epigenetic modifications mechanistically mediate the observed enhanced effector functions and bactericidal activity of IFN-γ–trained MDM.

### IFN-γ training enhanced cell surface marker expression and cytokine responses from people who are genetically more susceptible to infections.

To progress our findings toward clinical utility, we sought to identify subpopulations who may benefit from trained immunity in high-risk settings, such as during outbreak scenarios or before surgery to mitigate the risk of postoperative infection. The *TIRAP* gene encodes the signaling adaptor protein Mal, which plays roles in both TLR and IFNGR signaling (11, 50, 51). The Ser180Leu, *TIRAP* rs8177374 SNP mediates susceptibility to infection. The heterozygous (SL) form of the polymorphism has been associated with host resistance to infection, while the homozygous (LL) form of the polymorphism has been associated with vulnerability to infection and sepsis (11, 24, 26, 52, 53). We hypothesized that inducing trained immunity with IFN-γ in monocytes from LL homozygotes would restore antibacterial immunity to levels comparable to their protected heterozygous counterparts.

**Figure 6:**
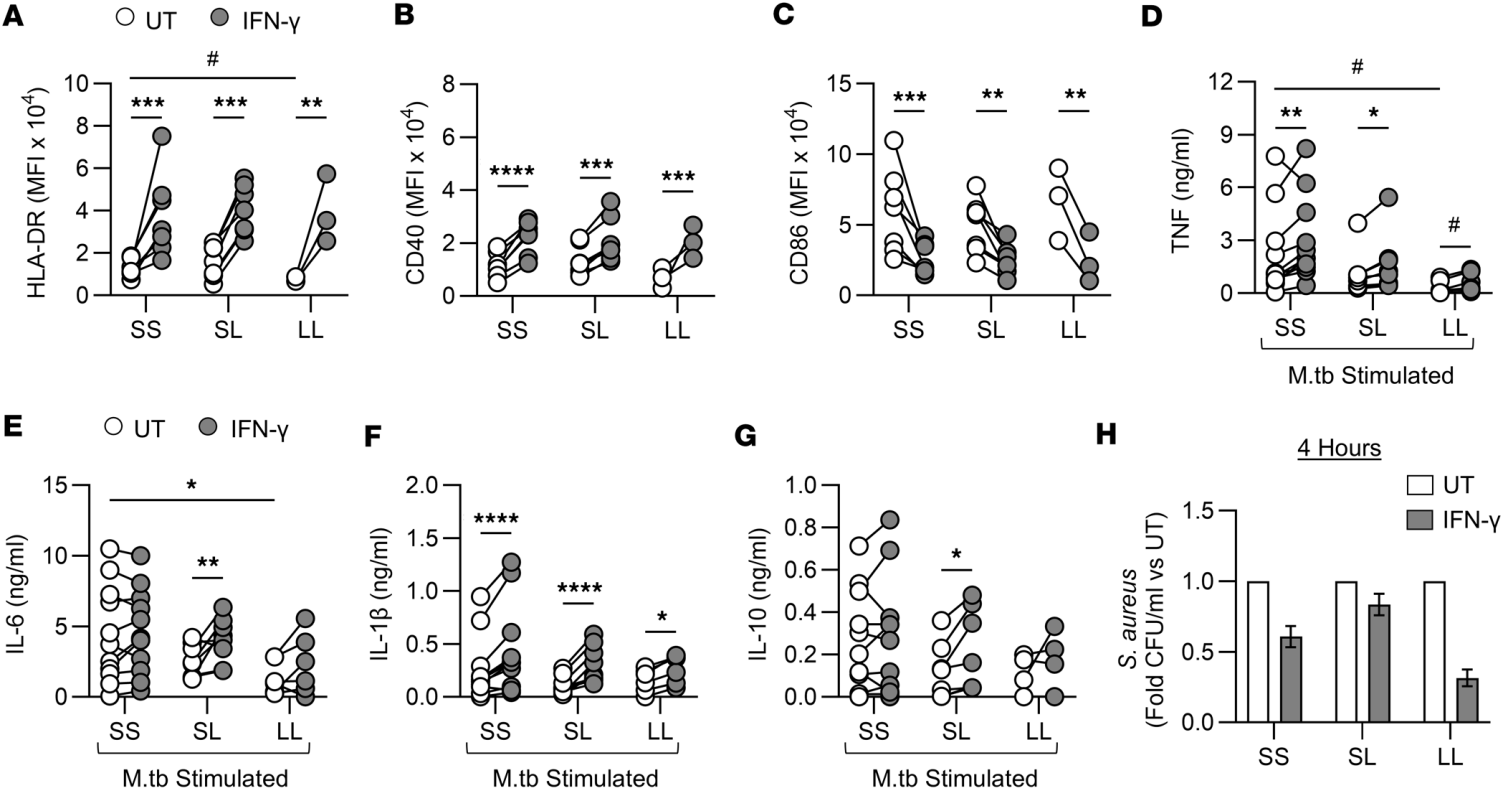
IFN-γ–induced trained immunity in donors with all *TIRAP* S180L polymorphism genotypes and enhanced killing of *S*. *aureus*. Enriched monocytes from donors with different genotypes of the *TIRAP* S180L polymorphism were left untrained (UT; white) or trained with IFN-γ (10 ng/mL; gray) for 24 hours. Cells were differentiated into MDM. (**D**–**G**) On day 6, MDM were stimulated with irradiated M.tb (10 μg/mL) for 24 hours. (**H**) On day 7, MDM were infected with *S*. *aureus* (USA300; MOI 100:1) for 4 hours, and bacterial burden was measured by CFU enumeration. (**A**–**C**) Expression of HLA-DR (**A**), CD40 (**B**), or CD86 (**C**) on unstimulated MDM of day 7 measured by flow cytometry grouped by *TIRAP* genotype. (**D**–**G**) TNF (**D**), IL-6 (**E**), IL-1β (**F**), or IL-10 (**G**) measured by ELISA grouped by genotype. (**H**) The intracellular bacterial burden of *S*. *aureus* within MDM was measured by CFU enumeration and grouped by *TIRAP* genotype graphed (compared with untrained MDM). *TIRAP* S180–homozygous (wild-type; SS, *n* = 11), S180L–heterozygous (SL, *n* = 8), or S180L–homozygous (LL, *n* = 6) individuals are plotted with each dot representing a single donor and paired data joined by a line. (**H**) Data show *n* = 3 (*n* = 1 SS, *n* = 1 SL, *n* = 1 LL). *P < 0.05, **P < 0.01, ***P < 0.001, ****P < 0.0001 determined using a 2-way ANOVA with Tukey’s multiple-comparison test. (**A** and **D**) Data were analyzed by 2-way ANOVA; however, results were not statistically significant. ^#^*P* < 0.05, paired *t* test comparing SS UT MDM with LL UT MDM (**A**), or SS UT MDM stimulated with M.tb with LL UT MDM stimulated with M.tb (**D**).

Blood donors were genotyped for *TIRAP* S180L; SS (wild-type; *n* = 11), SL (*n* = 8), and LL (*n* = 6), and training assays were performed. Expression of cell surface markers on donor MDM was determined by flow cytometry on day 7 (Figure 6, A–C, and Supplemental Figure 7, A–G). HLA-DR expression in untrained MDM was significantly reduced in LL MDM compared with SS MDM (Figure 6A). IFN-γ–trained MDM showed significantly enhanced HLA-DR and CD40 (Figure 6, A and B) and significantly reduced CD86 (Figure 6C) in all genotypes. This was maintained after MDM were stimulated with M.tb (Supplemental Figure 7, C–G).

MDM were stimulated with M.tb, and cytokine production was quantified by ELISA (Figure 6, D–G). LL MDM had significantly reduced production of TNF and IL-6 in response to M.tb compared with SS MDM (Figure 6, D and E). IFN-γ training significantly boosted TNF and IL-1β production in all genotypes compared with their own untrained controls (Figure 6, D and F). These data indicate that trained immunity restored cytokine production from LL MDM to levels comparable to those from protected SL MDM. Interestingly, SS MDM exhibited high IL-6 production, which did not further upregulate when monocytes were trained with IFN-γ, whereas LL MDM produced significantly less IL-6 in response to stimulation with M.tb. IFN-γ training did not significantly enhance IL-6 production from LL MDM (Figure 6E). SL MDM significantly increased IL-6 and IL-10 production in response to stimulation (Figure 6, E and G).

### IFN-γ training supported immunity to MRSA in MDM with the homozygous TIRAP 180L mutation.

We infected trained and untrained MDM from SS, SL, and LL donors with MRSA; IFN-γ training reduced CFU in all genotypes, with the most profound effect observed in LL MDM (Figure 6H). Collectively, these data indicate that inducing trained immunity in people who are vulnerable to infection, such as *TIRAP* 180L homozygotes, may enhance antibacterial immunity to pathogens of concern.

## Discussion

The central role of IFN-γ is established in many settings of trained immunity in vivo, including BCG, influenza A virus, and adenoviral vectors (8, 10, 19, 20). Our work shows that IFN-γ alone is sufficient to induce trained immunity in human monocytes in vitro, mediated by metabolic and epigenetic reprogramming. Inhibiting features of cellular metabolism or histone methylation during the training phase abrogated the enhanced production of IL-6 from MDM in response to subsequent bacterial challenge. Interestingly, inhibiting glycolysis with 2DG during the training phase did not inhibit this enhancement of IL-6. However, glycolysis was significantly enhanced in IFN-γ–trained MDM upon bacterial challenge, suggesting that the shift toward glycolytic metabolism results from metabolic reprogramming that does not rely on glycolysis for its induction. Furthermore, there was no increased chromatin accessibility in cellular pathways associated with glycolysis in our ATAC-sequencing dataset. These findings were unexpected, as glycolysis is widely reported to be essential for the induction of trained immunity (3, 5) and IFN-γ activated mTORC1, a known driver of glycolysis. Glycolysis is important for cytokine production (17, 39, 54), bacterial killing (18, 39), and ROS production (55), suggesting that different pathways are activated during the secondary challenge that are distinct from the initial metabolic reprogramming induced by IFN-γ.

Inhibiting glutaminolysis significantly attenuated training in our model, demonstrating its critical role in inducing trained immunity. Furthermore, inhibiting glutaminolysis or the conversion of succinate to fumarate inhibited H3K27ac. This aligns with the induction of trained immunity by β-glucan, mediated by glutaminolysis, with fumarate accumulation identified as driving H3K4me3 and H3K27ac (4). Inhibiting the conversion of fumarate to malate, resulting in the buildup of fumarate within the cell, induced a trained-like state in untrained MDM and enhanced IL-6 production after stimulation. These findings suggest that fumarate accumulation is important for IFN-γ training. Inhibiting histone methylation during IFN-γ training impeded enhanced IL-6 production from MDM, suggesting that early methylation events are crucial to induce the functional effects of trained immunity. Inhibiting FAO in untrained MDM with etomoxir resulted in enhanced IL-6 production in untrained cells, similarly to FHIN1, suggesting that fatty acid accumulation may be involved in IFN-γ–induced trained immunity. Moreover, ATAC-sequencing demonstrated increased chromatin accessibility in regions associated with lipid metabolism, and further research is needed to investigate this link.

IFN-γ–trained MDM exhibited a new baseline, defined by increased expression of cell surface markers and elevated gene expression of inflammatory regulators and glycolytic enzymes in comparison with untrained MDM. This is in keeping with findings from myeloid cells trained with β-glucan, BCG, or adenoviral vectors (1, 10, 27, 56). However, IFN-γ–trained MDM do not exhibit any change in cytokine and chemokine production in the absence of a stimulus and do not have altered baseline cellular metabolism, suggesting that these cells return to a functional homeostasis during the resting period. Thus, changes induced by IFN-γ training occur as a result of a new phenotypic baseline and not because they are primed or activated.

Altered capacity to produce cytokines and chemokines is the key functional output of trained immunity that mediates protection from subsequent challenges (10, 27, 57). While IL-6, TNF, and IL-1β secretion is often enhanced in cells that have undergone proinflammatory training (1, 2, 9, 58), IL-10 has been reported to be increased in LPS-tolerized cells (56, 59) and in cells trained with antiinflammatory *Fasciola hepatica* secretory products (60) or β-glucan (61). Thus, increases in IL-10 production in this study using the IFN-γ training model are unexpected; however, an increase in IL-10 was observed in trained monocytes following ChAdOx1 nCoV-19 vaccination (27). These findings align with observations in primary human macrophages where IFN-γ priming and/or bacterial stimulation upregulates both proinflammatory mediators and IL-10 concomitantly (17, 62).

*IRF1* is essential for IFN-γ–dependent macrophage immunity to mycobacteria, mediated through STAT1 signaling (63). Our data demonstrated that IFN-γ training increased expression of *IRF1* and enzymes associated with the NADPH oxidase system: *CYBB*, *NCF1*, and *NCF2*. We have also demonstrated increased ROS production from IFN-γ–trained MDM challenged with M.tb or MRSA. ATAC-sequencing supports these observations, demonstrating increased chromatin accessibility in pathways associated with ROS production. While these data are consistent with other training agents that also enhance ROS production (61, 64–66), to our knowledge, our data provide the first evidence that human IFN-γ–trained monocytes elicit MDM with enhanced bactericidal capacity mediated at least in part by ROS.

IFN-γ–mediated trained immunity resulted in protection against subsequent *Streptococcus* *pneumoniae* infection in an animal model due to enhanced neutrophil recruitment (10). In vivo, we postulate that IFN-γ–trained monocytes and macrophages will propagate immunity against subsequent challenge through a network of innate and adaptive immune recruitment and responses. IFN-γ has been shown to modulate hematopoiesis (67) and myelopoiesis during bacterial infections in vivo (68), and therefore it may enhance myelopoiesis and induce trained immunity within the bone marrow, similarly to BCG vaccination (8). Our reductive model shows that trained MDM are sufficient to enhance antibacterial immunity in vitro. While these reductions in bacterial burden were modest, they suggest that trained immunity could be combined with existing antimicrobial regimens to aid pathogen killing. Furthermore, we demonstrated that the enhanced proinflammatory cytokines and chemokines produced by IFN-γ–trained MDM are positively correlated with bactericidal capacity. Given the time frame in which this enhanced bactericidal capacity is evident, and the experimental monoculture, this bactericidal capacity is likely due to early mediators such as ROS. Inhibiting ROS production abrogated the effect of training on bacterial killing, suggesting that this is attributable to enhanced ROS production. To further support this hypothesis, we trained monocytes from a patient with CGD with a LOF mutation in *CYBB* resulting in an inability to produce ROS. Notably, monocytes carrying this mutation were trained by IFN-γ, resulting in increased IL-6 and TNF production after MRSA infection, but no IL-1β was detected, supporting the association between ROS production and IL-1β secretion (45, 46, 69, 70). The LOF in the NADPH oxidase system abrogated the enhanced bactericidal effect of trained immunity induced by IFN-γ. Cumulatively, these data indicate that ROS production, and associated IL-1β production, may mechanistically mediate the bactericidal effects of IFN-γ–induced trained immunity (45, 46, 68–70).

Trained immunity has deepened our understanding of the innate immune system and highlighted the salience of innate immune function in mediating protective immunity in vulnerable populations, such as neonates (71). However, translating trained immunity into clinical benefit is challenging; for example, BCG-induced trained immunity failed to protect against COVID-19 (72). Therefore, we sought to define the effects of IFN-γ training in healthy people with genetic vulnerability to infectious diseases who may be disproportionately at risk in outbreak scenarios. Our data indicate that macrophages from people who are homozygous for the 180L SNP in *TIRAP* express significantly less HLA-DR compared with macrophages from people with the wild-type genotype. Low HLA-DR on monocytes is a risk factor for mortality from sepsis, while elevated HLA-DR is a predictive marker for survival in severe sepsis (73–75). In addition, macrophages from homozygous LL donors produce significantly less TNF and IL-6 in response to challenge with M.tb, corroborating previous findings (11). Interestingly, IFN-γ trained all genotypes, resulting in significantly enhanced expression of HLA-DR and CD40, and production of TNF and IL-1β. Training MDM from homozygous LL donors boosted immunity to levels comparable to those seen with MDM from protected heterozygotes. Interestingly, MRSA killing by MDM differed by *TIRAP* 180L genotype, with a 39.5%, 17%, and 69% reduction in the wild-type, protected heterozygotes, and susceptible homozygotes, respectively (Figure 6H). These data demonstrate that while all genotypes had enhanced bacterial killing following IFN-γ training, the susceptible homozygotes had the greatest increase in bacterial killing, and the protected heterozygotes had only a modest increase. While IFN-γ–trained macrophages from all genotypes enhanced their TNF and IL-1β production in response to M.tb, only heterozygous macrophages increased IL-6 production. This enhanced proinflammatory function in protected heterozygous donors following training may potentially result in pathogenic effects. Therefore, studying different susceptible populations will allow us to identify and target vulnerable cohorts who may benefit from training. This allows a more targeted approach to induce training therapeutically and thus limit the potentially pathogenic effects of increased inflammation from trained immunity (28, 76).

Our work may also have clinical utility for patients experiencing immunoparalysis characterized by low HLA-DR expression on monocytes, such as those with severe tuberculosis (TB) or TB sepsis (77, 78). This phenotype is linked to metabolic defects and is associated with immunoparalysis in sepsis (79). Using training to boost HLA-DR expression may restore monocyte function in these patients. Furthermore, inducing trained immunity in patients with *S*. *aureus* bacteremia or severe TB marked by low HLA-DR may mitigate the risk of developing immunoparalysis and sepsis, and warrants clinical investigation. Ongoing IFN-based therapies are not well tolerated; however, our work suggests that a single dose may be efficacious to mediate short-term protection for vulnerable people in high-risk settings. Further studies are warranted to determine whether IFN-γ can reach the bone marrow to induce training in the longer term, and whether IFN-γ–exposed monocytes retain their trained immunity to infection in tissues, especially at mucosal sites. In addition, defining the effects of IFN-γ training in human tissue-resident macrophages such as the alveolar macrophages in lungs will aid the design of respiratory mucosal immunization strategies.

In conclusion, we provide evidence to support the hypothesis that exposing human monocytes to IFN-γ induces trained immunity in macrophages by enhancing host responses to infection with M.tb or MRSA. Moreover, a single exposure of IFN-γ to monocytes from genetically vulnerable people was sufficient to support macrophage immunity to these pathogens of concern. These data have important implications for the design of immunosupportive host-directed adjunctive therapy or preemptive prophylactic interventions in vulnerable or immunosuppressed hosts.

## Methods

### Sex as a biological variable.

Our study involved male and female participants. For experiments using buffy coats from the Irish Blood Transfusion Service (IBTS), sex of donor is unavailable. For experiments using blood collected from healthy volunteers to assess the ability of IFN-γ to train donors with different *TIRAP* genotypes, 13 participants (52%) were female and 12 were male (48%). This includes 5 (45%) SS donors, 5 (62.5%) SL donors, and 3 (50%) LL donors. We found no significant differences in our results when sex was considered.

### Participant recruitment.

Buffy coats from healthy donors were obtained with consent from the IBTS. Whole blood was collected with written informed consent from healthy volunteers and patients from the outpatient department of St. James’s Hospital Dublin.

### Monocyte in vitro training assays.

Peripheral blood mononuclear cells (PBMC) were isolated from buffy coats or whole blood by density centrifugation over Lymphoprep (STEMCELL Technologies). Cells were washed twice in PBS, and residual platelets were removed by centrifugation. PBMC were resuspended in 3 mL serum-free RPMI (Gibco GlutaMAX) and layered onto 10 mL of hyperosmotic Percoll solution (GE Healthcare; 48.5% Percoll, 41.5% sterile H_2_O, 0.16 M NaCl) to enrich for monocytes. Cells (2 × 10^6^ cells/mL) were plated on non-treated plates (Costar) and placed in a humidified incubator at 37°C, 5% CO_2_ for 1 hour to allow monocyte adherence. Non-adherent cells were removed by washing twice with PBS. Cells were cultured in RPMI supplemented with 10% type AB human serum (Sigma-Aldrich, H4522). Monocytes were left untrained or trained with IFN-γ (10 ng/mL; BioLegend, 570206) for 24 hours. Cells were washed with PBS, to remove IFN-γ, and incubated for 5 days to allow monocytes to differentiate into macrophages (MDM) with medium changes on days 2 and 5. For cell stimulation assays, MDM were stimulated on day 6 with LPS (10 ng/mL; Sigma-Aldrich, L5418) or irradiated *Mycobacterium tuberculosis* strain H37Rv (M.tb) for 24 hours.

### Preparation of irradiated M. tuberculosis.

M.tb, a gift from BEI Resources (NR-49098), was passed through a 23-gauge needle 8 times, and large clumps were removed by centrifugation. The supernatant containing suspended bacteria was collected. Protein concentration was measured using the Pierce BCA Protein Assay Kit (Thermo Fisher Scientific, 23225), following the manufacturer’s instructions.

### Infection of MDM.

*M*. *tuberculosis* H37Rv (ATCC, 27294) was propagated to log phase in Middlebrook 7H9 medium supplemented with ADC (BD, 211887). MDM were infected as previously described (80).

### S.

*aureus* (LAC USA300) was cultivated from frozen stocks, streaked onto tryptic soy agar (TSA) plates, and incubated overnight at 37°C. Bacterial colonies were suspended in PBS and adjusted to an optical density (OD) of 1 corresponding to 1 × 10^9^ CFU/mL. MDM were infected with *S*. *aureus* (MOI 100:1) and incubated for 1 hour. MDM were washed with PBS to remove extracellular bacteria, treated with gentamicin (100 μg/mL; Sigma-Aldrich, G1397), and incubated for indicated times. Cells were lysed with 0.1% Triton X-100 (Sigma-Aldrich, X100), lysates were serially diluted, plated on TSA plates, and incubated overnight, and CFU were counted after 24 hours.

### Crystal violet assay for cellular density and viability.

Cell supernatants were removed, and cells were fixed with 2% paraformaldehyde for 20 minutes. After 2 PBS washes, 0.1% crystal violet (CV; Sigma-Aldrich) was added, and plates were incubated for 30 minutes at room temperature, washed with PBS, and air-dried. CV was dissolved with 0.1% Triton X-100, and absorbance was measured at 600 nm. Cell viability was assessed based on OD, with values used to estimate relative cell density. Viability was expressed as fold change relative to control (untrained, unstimulated), calculated by division of each sample’s OD by the control.

### Flow cytometry.

MDM were placed in ice-cold PBS and incubated at 4°C on ice for 30 minutes. Cells were removed by gentle scraping, Fc-blocked with Human TruStain FcX (BioLegend, 422302), and stained with Zombie NIR viability dye (BioLegend, 423106) and fluorochrome-conjugated antibodies specific for CD14 (AF488; clone HCD14; BioLegend, 325610), CD68 (PE; clone Y1/82A; BioLegend, 333808), CD83 (PerCP-Cy5.5; clone HB15e; BioLegend, 305320), CD80 (PE-Cy7; clone 2D10; BioLegend, 305218), HLA-DR (APC; clone L243; BioLegend, 307610), CD86 (BV421; clone IT2.2; BioLegend, 305426), CD40 (BV510; clone 5C3; BioLegend 334330), TLR2 (APC; clone TL2.1; BioLegend, 309720), or TLR4 (APC; clone HTA125; BioLegend, 312816). If surface staining only, cells were fixed with 2% paraformaldehyde. If staining intranuclearly for H3K27ac (AF647; clone EP16602; Abcam, ab245912) or H3K4me3 (PE; clone EPR20551-225; Abcam, ab237342), the FOXP3 Staining Buffer Set (eBioscience, 00-5523-00) was used following the manufacturer’s instructions. Samples were acquired on a BD FACSCanto II or BD LSRFortessa.

For ROS detection, MDM were loaded with 2.5 μg/mL dihydrorhodamine-123 (DHR123; Invitrogen, 11510346) (M.tb only) and 5 μg/mL cytochalasin B (Sigma-Aldrich, C2743) for 10 minutes at 37°C. Cells were stimulated with irradiated *M*. *tuberculosis* (10 μg/mL) or infected with *S*. *aureus* (MOI 100:1) for 4 hours. Cells were washed with PBS and fixed in 2% paraformaldehyde. To assess mitochondrial ROS, cells were stimulated with irradiated *M*. *tuberculosis* (10–20 μg/mL) for 6 hours. Cells were stained with 2.5 μM MitoSOX Red (Invitrogen, M36008) for 20 minutes at 37°C in RPMI. Cells were washed with PBS. All ROS assays were immediately analyzed on a BD FACSCanto II or Cytek Northern Lights.

CFSE-labeled *S*. *aureus* was used to determine bacterial uptake by MDM. *S*. *aureus* (1 × 10^9^ CFU) was labeled with 5 μM CFSE (Thermo Fisher Scientific, C34554) by incubation on a rotator at 37°C for 30 minutes. Labeled bacteria were washed 3 times with PBS and resuspended in RPMI supplemented with 10% FBS (Sigma-Aldrich). MDM were infected with CFSE-labeled *S*. *aureus* (MOI 100:1) for 1 hour at 37°C. After washing with PBS, cells were detached as above, fixed with 2% paraformaldehyde, and acquired on a BD FACSCanto II. Bacterial uptake was measured as the frequency of CFSE^+^ MDM, and bacterial load was quantified by median fluorescence intensity. Macrophage phagocytosis was measured using Latex Beads (Cambridge Bioscience, CAY500290; 1:400 dilution) for 50 minutes at 37°C. Cells were then washed, fixed, and acquired on the LSRFortessa or Northern Lights.

Cell surface marker expression, ROS production, and phagocytosis were measured using median fluorescence intensity values. Unstained cells and fluorescence minus one controls were used to normalize for background staining and to set gates. Data were analyzed using FlowJo software v10.10 (Becton Dickinson).

### Cytokine measurement.

The concentrations of IL-1β (BioLegend, 437016), IL-10 (BioLegend, 430604), IL-6 (BioLegend, 430516), IFN-γ (BioLegend, 430116), TNF (Invitrogen), MIP-1α (R&D Systems, DY270), and CXCL1 (R&D Systems, DY275) were determined by ELISA following each manufacturer’s instructions. A Human Luminex Discovery Kit (Bio-Techne, LXSAHM-18) was designed to assess analytes in *S*. *aureus*–infected supernatants: TNF, IL-6, IL-1β, IL-10, GM-CSF, G-CSF, IL-1α, IL-1Ra, IL-23, CXCL10, MCP-1, MIP-1α, MIP-1β, CXCL1, CXCL2, CXCL9, CXCL10, and CCL1. The assay was carried out per the manufacturer’s protocol and analyzed on a Luminex MAGPIX System.

### qPCR.

RNA was extracted (RNeasy Mini Kit, QIAGEN, 74136) following the manufacturer’s instructions. RNA content and quality were quantified using a NanoDrop spectrophotometer (Thermo Fisher Scientific), and RNA was reverse-transcribed using SensiFast Reverse Transcription Kit (Meridian Biosciences, BIO-65054). Catalogued TaqMan (Thermo Fisher Scientific) predesigned primer probes were used and duplexed with *18S* as an endogenous control (Hs03003631_g1: VIC). The following probes were used (TaqMan: FAM): *ATP5B* (Hs00969569_m1), *CYBA* (Hs00609145_m1), *CYBB* (Hs00166163_m1), *GAPDH* (Hs02786624_g1), *GPI* (Hs00976715_m1), *HK1* (Hs00175976_m1), *IRF1* (Hs00971965_m1), *NCF1* (Hs00417167_m1), *NCF2* (Hs01084940_m1), *NFKB1* (Hs00765730_m1), *PFKFB3* (Hs00998698_m1), *PKM2* (Hs00761782_s1), and *TBX21* (Hs00894392_m1). qPCR was performed using SensiFast Probe Hi-ROX Kit (Meridian Biosciences, BIO-82005) on a QuantStudio 5 Real-Time qPCR System (Applied Biosystems). Relative quantitative data were obtained and analyzed using the 2^–ΔΔCt^ method. Gene expression was calculated relative to untrained MDM sample.

### Immunoblotting for phospho-S6.

Monocytes were magnetically isolated from PBMC using the EasySep Human CD14 Positive Selection Kit (STEMCELL Technologies, 17858) according to the manufacturer’s instructions. Monocytes (1 × 10^6^ cells/mL) were treated with IFN-γ (10 ng/mL) for 15 minutes alone or with rapamycin (50 nM; Sigma-Aldrich, R8781) and lysed with 1× Laemmli buffer (containing 20% β-mercaptoethanol). Lysates were then analyzed as previously described (80) and detected for phospho-S6 (Cell Signaling Technology, 2211; 1:1,000).

### Metabolic assays using the Seahorse XFe analyzer.

MDM extracellular flux analysis was performed using the Seahorse XFe24 Analyzer (Agilent). MDM (1 x 10^5^) were seeded into wells on a Seahorse plate. The next day, 10× concentrations of LPS (10 ng/mL; Sigma-Aldrich, L5418) or irradiated M.tb (10 μg/mL) or medium (unstimulated group; sham injection) were loaded into the ports on the cartridge. The ECAR and OCR were measured every 9 minutes to establish baseline rates. The cells were stimulated and monitored in real time. Medium, LPS, or irradiated M.tb was injected into assigned wells at approximately 18 minutes. Analysis was carried out using Wave Software (Agilent), and differences in cell number were normalized to CV values. ECAR and OCR readings were calculated relative to the control (untrained unstimulated) group.

### Metabolic inhibitor analysis.

Enriched monocytes were pretreated for 1 hour with rapamycin (50 nM; Sigma-Aldrich, R8781), 2DG (1 mM; Sigma-Aldrich, D6134), etomoxir (5 μM; Sigma-Aldrich, 236020), BPTES (50 μM; Sigma-Aldrich, SML0601), fumarate hydratase inhibitor 1 (10 μM; MedChemExpress, HY-100004), MTA (1 mM; Sigma-Aldrich, D5011), or dimethyl malonate (1 mM; Sigma-Aldrich, 136441). After inhibitor treatment, cells were stimulated with IFN-γ (10 ng/mL; BioLegend, 570206) and incubated for 24 hours. Cells were washed with warm PBS and cultured for 5 days. On day 6, MDM were stimulated with LPS (10 ng/mL; Sigma-Aldrich, L5418) or M.tb (10 μg/mL) for 24 hours. Supernatants were collected and stored at –20°C for quantification of IL-6.

### ATAC-sequencing.

ATAC-sequencing analysis was performed on a dataset from O’Farrell et al. (49). Human immune cells from 3 healthy anonymized human donors (ages 25, 30, and 60, all female) were collected from blood apheresis product by the Human Immunology Core at the Perelman School of Medicine at the University of Pennsylvania. Monocytes were isolated by negative selection using the RosetteSep (STEMCELL Technologies, 1506) kit and seeded at a density of 800,000 cells/mL in Cellvis 12-well glass plates. Monocytes were left untrained or trained with IFN-γ (50 ng/mL) for 24 hours. After 24 hours, wells were washed twice with PBS and cultured for an additional 5 days. As O’Farrell et al. used 50 ng/mL of IFN-γ to induce training, we assessed this higher training dose (50 ng/mL) of IFN-γ and found significantly enhanced IL-6 and IL-1β in response to M.tb (Supplemental Figure 8, A and B).

On day 6, we performed a modified form of the OMNI-ATAC sequencing protocol described in ref. 81 on untrained and IFN-γ–trained cells from 3 donors, collecting 100,000 cells per technical duplicate. Cells were lysed (10% NP-40, 10% Tween 20, and 1% digitonin) for 3 minutes at 4°C, then transposed with Tn5 (a gift from Kavitha Sarma, Wistar Institute, Philadelphia, Pennsylvania, USA) for 30 minutes at 37°C. Transposed genomic DNA was isolated using a Zymo DNA Clean and Concentrator kit and amplified using custom primers (originally designed by Greenleaf Lab, Stanford University, Stanford, California, USA) for 13 PCR cycles. We then performed double-sided purification and size selection with AMPure XP magnetic beads (Beckman Coulter) and confirmed final concentration with Agilent Bioanalyzer High Sensitivity DNA chips. Final libraries were sequenced with a P4 XLEAP 100-cycle kit on an Illumina NextSeq 2000, at depth of approximately 50 million paired-end reads per sample.

We first used Bowtie 2 (http://bowtie-bio.sourceforge.net/bowtie2/index.shtml) to align all sequencing reads to hg38, then removed PCR duplicates with Picard’s MarkDuplicates (http://broadinstitute.github.io/picard). We used the ATACseqQC package to clean BAM files and perform a Tn5 insertion shift.

We used MACS2’s (82) callpeak function to identify peaks, setting an FDR cutoff of 0.05, and inputting technical-replicate-merged samples filtered to a fragment size less than 300 bp to improve accuracy. We generated 1 non-redundant peak list, filtered to only include peaks present in at least 2 samples and not present on the ENCODE list (83). Finally, we used the summarizeOverlaps function from the GenomicAlignments package to generate a full counts matrix for all samples (unmerged and unfiltered by fragment size) across these peaks.

With this counts matrix, we identified differentially accessible regions of chromatin for each donor using DESeq2 (84), after setting a minimum threshold of 20 reads per peak. We selected regions deemed statistically significantly differentially accessible (*P*_adj_ < 0.05) from untrained cells in at least 2 of the 3 sequenced donors. We then grouped these selected regions into 4 categories — regions that opened with IFN-γ training (with log_2_ fold change [log_2_FC] > 0 in trained cells compared with untrained cells), regions that opened greatly with IFN-γ training (log_2_FC > 2), regions that closed with IFN-γ training (log_2_FC < 0), and regions that closed greatly with IFN-γ training (log_2_FC < –2). We performed gene ontology enrichment analysis compared against the full peak list for each of these 4 groups using GREAT (85). Selected statistically significant terms (FDR < 0.05) from this analysis can be found in Figure 5A; the full output tables can be found in the Supplemental Table 1.

For selected genes in Figure 5C, we identified all peaks present within the gene body of each gene of interest and calculated the log_2_FC in accessibility between trained and untrained cells at each peak for each donor using DESeq2. We then averaged the log_2_FC in accessibility across peaks within each gene of interest for each donor.

We used TOBIAS (86) to identify putative differentially bound transcription factors within accessible chromatin of trained and untrained cells. We first used the function ATACorrect to identify regions of protection from Tn5 cuts (suggestive of actively bound protein at that location), and ScoreBigWig to calculate footprint scores. Differentially enriched motifs at these footprints were identified using BINDetect. Highlighted differential factors were all those above the 95% quantile of –log_10_(*P* value) and/or above the 95% quantile and below the 5% quantile of differential binding scores. We further filtered this list to factors highlighted in at least 2 of our 3 donors and removed any redundant factors. In Figure 5B, we plot the mean differential binding score across the 3 donors for each factor in the filtered list.

For heatmaps in Figure 5, D–F, we normalized counts matrices by read depth calculating the counts per million for each peak, then merged technical replicates. For each gene of interest, we filtered to the highly confident differential peaks (*P*_adj_ < 0.05 in at least 2 of 3 donors, as discussed above) within the gene body. We averaged the counts per million across peaks for each gene, for each donor, and plotted as a row-scaled heatmap.

### Genotyping.

DNA isolation was performed on saliva or blood from consenting donors (DNeasy Blood and Tissue Kit, QIAGEN, 69504) following the manufacturer’s instructions. DNA concentration was determined using a NanoDrop spectrophotometer. Genotyping of the *TIRAP* 180L polymorphism was performed using a TaqPath ProAmp Master Mix (Applied Biosystems, A30865), and the TaqMan custom human SNP (SNP ID: rs8177374) genotyping assay (Applied Biosystems, 4362691) was performed following the manufacturer’s instructions. A non-template control (i.e., nuclease-free H_2_O) and positive control (i.e., a DNA sample of known genotype) were assayed in parallel. Training assays were performed using fresh venous blood from 25 genotyped donors (52% female, 48% male; median age 40) who provided informed consent (Table 1).

### Statistics.

Statistical analyses were performed using GraphPad Prism 10.4.2 software. Statistically significant differences between 2 normally distributed groups were determined using Student’s paired *t* tests with 2-tailed *P* values. Differences between 3 or more groups were determined by 1-way ANOVA with Tukey’s multiple-comparison test or with an uncorrected Fisher’s LSD test. Differences between 2 or more groups containing more than 1 variable were determined by 2-way ANOVA with Šidák’s multiple-comparison tests. *P* values of ≤ 0.05 were considered statistically significant and are denoted by asterisks. Where datasets containing 2 or more groups containing more than 1 variable were reanalyzed independently using paired *t* tests, *P* values of ≤ 0.05 are denoted with a hash mark. Details of specific tests performed can be found in the figure legends.

### Study approval.

The study was approved by the Tallaght University Hospital and St. James’s Hospital Joint Research Ethics Committee and by the Faculty of Health Science Research Ethics Committee, Trinity College Dublin. Whole blood was collected with written informed consent.

### Data availability.

Supporting data, including values for all data points shown in graphs, are available in the Supporting Data Values file.

## Author contributions

DMM contributed conceptualization, methodology, formal analysis, investigation, data curation, writing of the original draft of the manuscript, and visualization. IB contributed methodology, formal analysis, investigation, data curation, resources, review and editing of the manuscript, and visualization. AO, SRC, SAO, CC, BM, GL, GJ, SAC, AHD, JPM, EM, OST, AY, and CMF contributed methodology, formal analysis, investigation, and review and editing of the manuscript. CNC, JD, CÓM, LEG, AD, and NB contributed conceptualization, resources, and review and editing of the manuscript. DJC, NC, AR, and RMM contributed conceptualization, resources, formal analysis, investigation, and review and editing of the manuscript. RVC and JK contributed conceptualization, methodology, resources, formal analysis, funding acquisition, and review and editing of the manuscript. SAB contributed conceptualization, methodology, formal analysis, data curation, writing of the original draft of the manuscript, visualization, supervision, project administration, and funding acquisition.

## Funding support

- Health Research Board (EIA-2019-010 to SAB).
- Royal City of Dublin Hospital Trust (to JK).

## Supplementary Material

Supplemental data

Unedited blot and gel images

Supplemental table 1

Supporting data values

## Figures and Tables

**Table 1:**
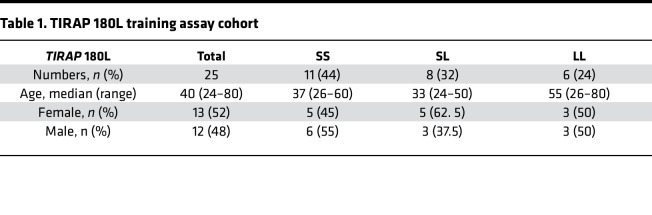
TIRAP 180L training assay cohort

## Version 1 02/10/2026

In-Press Preview

## Version 2 03/23/2026

Electronic publication

## References

[1] Quintin J (2012). *Candida albicans* infection affords protection against reinfection via functional reprogramming of monocytes. Cell Host Microbe.

[2] Kleinnijenhuis J (2012). Bacille Calmette-Guerin induces NOD2-dependent nonspecific protection from reinfection via epigenetic reprogramming of monocytes. Proc Natl Acad Sci U S A.

[3] Cheng SC (2014). MTOR- and HIF-1α-mediated aerobic glycolysis as metabolic basis for trained immunity. Science.

[4] Arts RJW (2016). Glutaminolysis and fumarate accumulation integrate immunometabolic and epigenetic programs in trained immunity. Cell Metab.

[5] Arts RJW (2016). Immunometabolic pathways in BCG-induced trained immunity. Cell Rep.

[6] Saeed S (2014). Epigenetic programming of monocyte-to-macrophage differentiation and trained innate immunity. Science.

[7] Keating ST (2020). Rewiring of glucose metabolism defines trained immunity induced by oxidized low-density lipoprotein. J Mol Med (Berl).

[8] Kaufmann E (2018). BCG educates hematopoietic stem cells to generate protective innate immunity against tuberculosis. Cell.

[9] Arts RJW (2018). BCG vaccination protects against experimental viral infection in humans through the induction of cytokines associated with trained immunity. Cell Host Microbe.

[10] Yao Y (2018). Induction of autonomous memory alveolar macrophages requires T cell help and is critical to trained immunity. Cell.

[11] Ní Cheallaigh C (2016). A common variant in the adaptor mal regulates interferon gamma signaling. Immunity.

[12] Wang QS (2018). Interferon-gamma induces autophagy-associated apoptosis through induction of cPLA2-dependent mitochondrial ROS generation in colorectal cancer cells. Biochem Biophys Res Commun.

[13] Wang F (2018). Interferon gamma induces reversible metabolic reprogramming of M1 macrophages to sustain cell viability and pro-inflammatory activity. EBioMedicine.

[14] Salim T (2016). Investigating the role of TNF-α and IFN-γ activation on the dynamics of iNOS gene expression in LPS stimulated macrophages. PLoS One.

[15] Spencer NG (2016). Mechanisms underlying interferon-γ-induced priming of microglial reactive oxygen species production. PLoS One.

[16] Braverman J (2016). HIF-1α is an essential mediator of IFN-γ-dependent immunity to Mycobacterium tuberculosis. J Immunol.

[17] Cox DJ (2024). Human airway macrophages are metabolically reprogrammed by IFN-γ resulting in glycolysis-dependent functional plasticity. Elife.

[18] Hackett EE (2020). Mycobacterium tuberculosis limits host glycolysis and IL-1β by restriction of PFK-M via microRNA-21. Cell Rep.

[19] Wang T (2023). Influenza-trained mucosal-resident alveolar macrophages confer long-term antitumor immunity in the lungs. Nat Immunol.

[20] Tran KA (2024). BCG immunization induces CX3CR1^hi^ effector memory T cells to provide cross-protection via IFN-γ-mediated trained immunity. Nat Immunol.

[21] Netea MG (2020). Defining trained immunity and its role in health and disease. Nat Rev Immunol.

[22] Mulder WJM (2019). Therapeutic targeting of trained immunity. Nat Rev Drug Discov.

[23] https://www.who.int/publications/i/item/9789240093461.

[24] Ferwerda B (2009). Functional and genetic evidence that the Mal/TIRAP allele variant 180L has been selected by providing protection against septic shock. Proc Natl Acad Sci U S A.

[25] Ladhani SN (2010). Association between single-nucleotide polymorphisms in Mal/TIRAP and interleukin-10 genes and susceptibility to invasive haemophilus influenzae serotype b infection in immunized children. Clin Infect Dis.

[26] Khor CC (2007). A Mal functional variant is associated with protection against invasive pneumococcal disease, bacteremia, malaria and tuberculosis. Nat Genet.

[27] Murphy DM (2023). Trained immunity is induced in humans after immunization with an adenoviral vector COVID-19 vaccine. J Clin Invest.

[28] Bekkering S (2014). Oxidized low-density lipoprotein induces long-term proinflammatory cytokine production and foam cell formation via epigenetic reprogramming of monocytes. Arterioscler Thromb Vasc Biol.

[29] Fanucchi S (2021). The intersection of epigenetics and metabolism in trained immunity. Immunity.

[30] Liu T (2017). NF-κB signaling in inflammation. Signal Transduc Target Ther.

[31] Ruffner H (1993). Induction of type I interferon genes and interferon-inducible genes in embryonal stem cells devoid of interferon regulatory factor 1. Proc Natl Acad Sci U S A.

[32] Pine R (1990). Purification and cloning of interferon-stimulated gene factor 2 (ISGF2): ISGF2 (IRF-1) can bind to the promoters of both beta interferon- and interferon-stimulated genes but is not a primary transcriptional activator of either. Mol Cell Biol.

[33] Feng H (2021). Interferon regulatory factor 1 (IRF1) and anti-pathogen innate immune responses. PLoS Pathog.

[34] Chow JC (1999). Toll-like receptor-4 mediates lipopolysaccharide-induced signal transduction. J Biol Chem.

[35] Sánchez D (2010). Role of TLR2- and TLR4-mediated signaling in Mycobacterium tuberculosis-induced macrophage death. Cell Immunol.

[36] Lighvani AA (2001). T-bet is rapidly induced by interferon-gamma in lymphoid and myeloid cells. Proc Natl Acad Sci U S A.

[37] Panwar V (2023). Multifaceted role of mTOR (mammalian target of rapamycin) signaling pathway in human health and disease. Signal Transduct Target Ther.

[38] Kaur S (2008). Dual regulatory roles of the phosphatidylinositol 3-kinase in interferon signaling. J Immunol.

[39] Gleeson LE (2016). Cutting edge: Mycobacterium tuberculosis induces aerobic glycolysis in human alveolar macrophages that is required for control of intracellular bacillary replication. J Immunol.

[40] Ó Maoldomhnaigh C (2021). The Warburg effect occurs rapidly in stimulated human adult but not umbilical cord blood derived macrophages. Front Immunol.

[41] Zahalka S (2022). Trained immunity of alveolar macrophages requires metabolic rewiring and type 1 interferon signaling. Mucosal Immunol.

[42] Groh LA (2021). oxLDL-induced trained immunity is dependent on mitochondrial metabolic reprogramming. Immunometabolism.

[43] Ferreira AV (2023). Fatty acid desaturation and lipoxygenase pathways support trained immunity. Nat Commun.

[44] Bustamante J (2011). Germline CYBB mutations that selectively affect macrophages in kindreds with X-linked predisposition to tuberculous mycobacterial disease. Nat Immunol.

[46] Li L (2020). ROS-mediated NLRP3 inflammasome activation participates in the response against Neospora caninum infection. Parasit Vectors.

[47] Ben-Ari J (2012). Infections associated with chronic granulomatous disease: linking genetics to phenotypic expression. Expert Rev Anti Infect Ther.

[48] Roos D (2016). Chronic granulomatous disease. Br Med Bull.

[50] Fitzgerald KA (2001). Mal (MyD88-adapter-like) is required for Toll-like receptor-4 signal transduction. Nature.

[51] Yamamoto M (2002). Essential role for TIRAP in activation of the signalling cascade shared by TLR2 and TLR4. Nature.

[52] Capparelli R (2013). The MyD88 rs6853 and TIRAP rs8177374 polymorphic sites are associated with resistance to human pulmonary tuberculosis. Genes Immun.

[53] Hamann L (2009). Low frequency of the TIRAP S180L polymorphism in Africa, and its potential role in malaria, sepsis, and leprosy. BMC Med Genet.

[54] Palsson-Mcdermott EM (2015). Pyruvate kinase M2 regulates Hif-1α activity and IL-1β induction and is a critical determinant of the Warburg effect in LPS-activated macrophages. Cell Metab.

[55] Toller-Kawahisa JE (2023). The metabolic function of pyruvate kinase M2 regulates reactive oxygen species production and microbial killing by neutrophils. Nat Commun.

[56] Jeljeli M (2019). Trained immunity modulates inflammation-induced fibrosis. Nat Commun.

[57] D’Agostino MR (2020). Airway macrophages mediate mucosal vaccine-induced trained innate immunity against *Mycobacterium tuberculosis* in early stages of infection. J Immunol.

[58] Moorlag SJCFM (2020). β-glucan induces protective trained immunity against Mycobacterium tuberculosis infection: a key role for IL-1. Cell Rep.

[59] Frankenberger M (1995). Interleukin-10 is upregulated in LPS tolerance. J Inflamm.

[60] Quinn SM (2019). Anti-inflammatory trained immunity mediated by Helminth products attenuates the induction of T cell-mediated autoimmune disease. Front Immunol.

[61] Bekkering S (2016). In vitro experimental model of trained innate immunity in human primary monocytes. Clin Vaccine Immunol.

[62] Carlile SR (2024). *Staphylococcus aureus* induced trained immunity in macrophages confers heterologous protection against Gram-negative bacterial infection. iScience.

[63] Rosain J (2023). Human IRF1 governs macrophagic IFN-γ immunity to mycobacteria. Cell.

[64] Ferreira AV (2021). Glutathione metabolism contributes to the induction of trained immunity. Cells.

[65] Sohrabi Y (2019). mTOR-dependent oxidative stress regulates oxLDL-induced trained innate immunity in human monocytes. Front Immunol.

[66] van der Heijden C (2020). Aldosterone induces trained immunity: the role of fatty acid synthesis. Cardiovasc Res.

[67] De Bruin AM (2014). Impact of interferon-γ on hematopoiesis. Blood.

[68] MacNamara KC (2011). Infection-induced myelopoiesis during intracellular bacterial infection is critically dependent upon IFN-γ signaling. J Immunol.

[69] Bulua AC (2011). Mitochondrial reactive oxygen species promote production of proinflammatory cytokines and are elevated in TNFR1-associated periodic syndrome (TRAPS). J Exp Med.

[70] Zhou R (2010). Thioredoxin-interacting protein links oxidative stress to inflammasome activation. Nat Immunol.

[71] Hong M (2015). Trained immunity in newborn infants of HBV-infected mothers. Nat Commun.

[72] Pittet LF (2023). Randomized trial of BCG vaccine to protect against Covid-19 in health care workers. N Engl J Med.

[73] Lekkou A (2004). Cytokine production and monocyte HLA-DR expression as predictors of outcome for patients with community-acquired severe infections. Clin Diagn Lab Immunol.

[74] Winkler MS (2017). Human leucocyte antigen (HLA-DR) gene expression is reduced in sepsis and correlates with impaired TNFα response: a diagnostic tool for immunosuppression?. PLoS One.

[75] Coakley JD (2019). Dysregulated T helper type 1 (Th1) and Th17 responses in elderly hospitalised patients with infection and sepsis. PLoS One.

[76] Arts RJW (2018). The potential role of trained immunity in autoimmune and autoinflammatory disorders. Front Immunol.

[77] Leventogiannis K (2022). Toward personalized immunotherapy in sepsis: the PROVIDE randomized clinical trial. Cell Rep Med.

[78] Brandes-Leibovitz R (2024). Sepsis pathogenesis and outcome are shaped by the balance between the transcriptional states of systemic inflammation and antimicrobial response. Cell Rep Med.

[79] Cheng SC (2016). Broad defects in the energy metabolism of leukocytes underlie immunoparalysis in sepsis. Nat Immunol.

[80] Ó Maoldomhnaigh C (2021). Lactate alters metabolism in human macrophages and improves their ability to kill *Mycobacterium tuberculosis*. Front Immunol.

[81] Corces MR (2017). An improved ATAC-seq protocol reduces background and enables interrogation of frozen tissues. Nat Methods.

[82] Zhang Y (2008). Model-based analysis of ChIP-Seq (MACS). Genome Biol.

[83] Amemiya HM (2019). The ENCODE blacklist: Identification of problematic regions of the genome. Sci Rep.

[84] Love MI (2014). Moderated estimation of fold change and dispersion for RNA-seq data with DESeq2. Genome Biol.

[85] McLean CY (2010). GREAT improves functional interpretation of cis-regulatory regions. Nat Biotechnol.

[86] Bentsen M (2020). ATAC-seq footprinting unravels kinetics of transcription factor binding during zygotic genome activation. Nat Commun.

