# Supplementary material for: IFN-γ–induced trained immunity enhances killing of priority pathogens in healthy and genetically vulnerable individuals
Source: JCI Insight. 2026 Feb 10;11(6):e195866. doi: 10.1172/jci.insight.195866 (PMC13043095; doi:10.1172/jci.insight.195866)
Supplement: Supplemental data [file jciinsight-11-195866-s064.pdf]

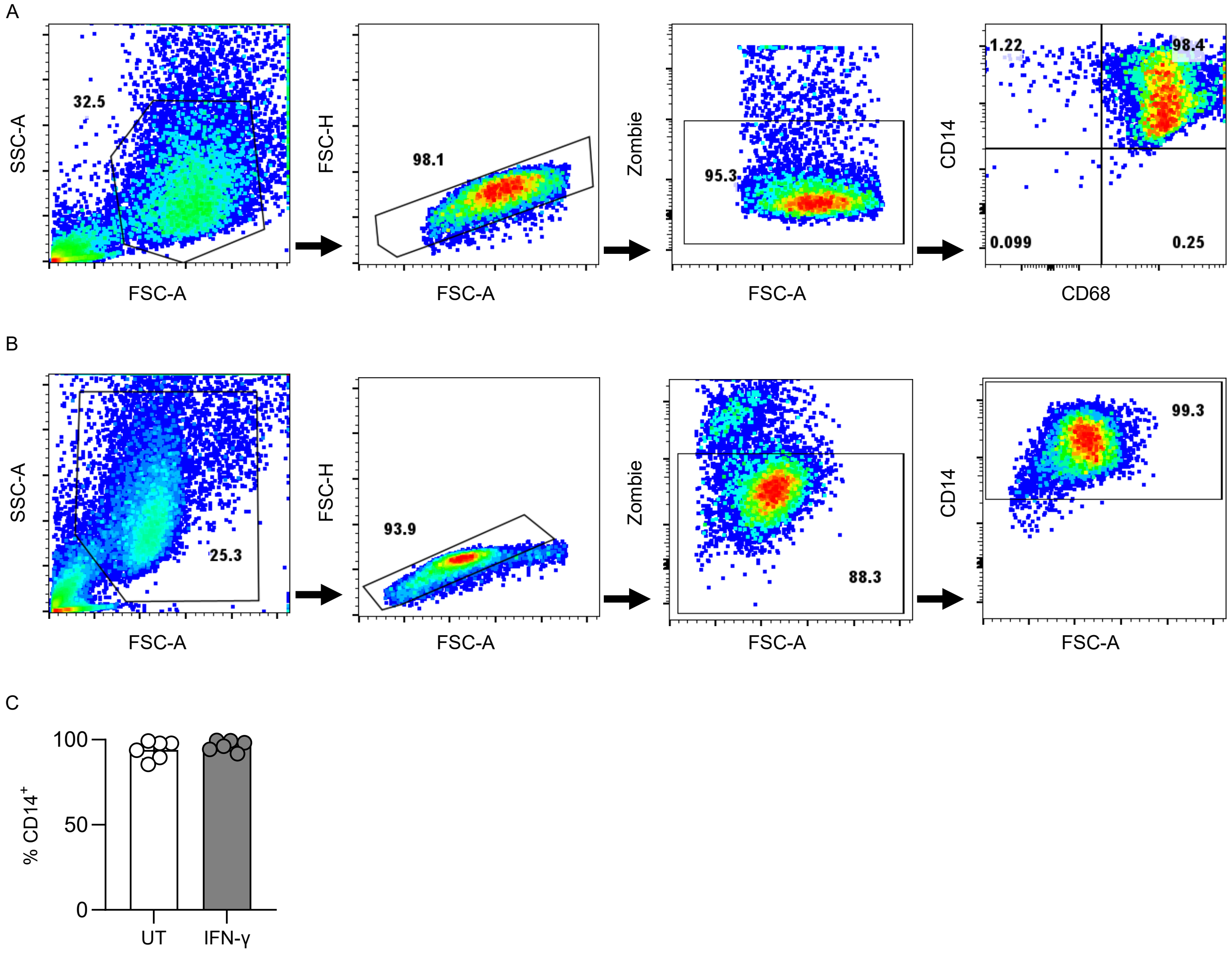

Supplemental Figure 1:
Human monocytes were enriched from PBMC using a hyperosmotic percoll gradient and plastic adherence. Enriched monocytes were left untrained (UT: white) or were trained with IFN-γ (10 ng/ml: grey) for 24 hours. Cells were then washed and matured into MDM. (A) Gating strategy for assessing MDM purity on day 7 by gating on cells, single cells, live cells and CD14$^+$ CD68$^+$ cells. (B) Gating strategy for assessing changes in MDM phenotype by gating on cells, single cells, live cells and CD14$^+$ cells. (C) The frequency of CD14 positive cells in UT and IFN-γ trained MDM cultures. Each dot represents an individual donor n=5.

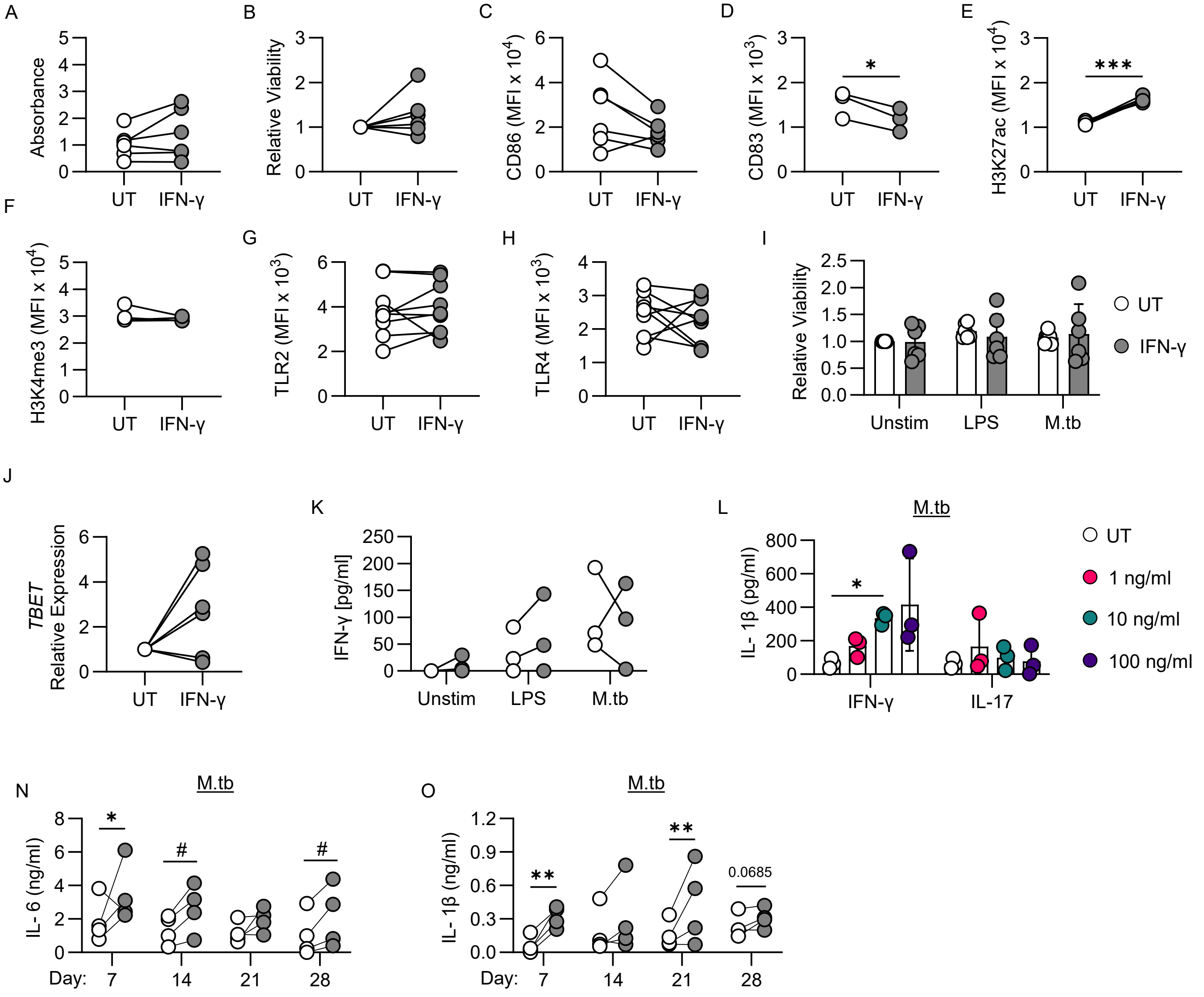

Supplemental Figure 2:
(A-K) Enriched monocytes were left untrained (UT: white) or were trained with IFN-γ (10 ng/ml: grey) for 24 hours. Cells were then differentiated into MDM. (A-B) Crystal violet staining measured by absorbance at 600 nm. (C-H) Expression of (C) CD86, (D) CD83, (E) H3K27ac, (F) H3K4me3, (G) TLR2, or (H) TLR4 on unstimulated MDM on day 7 was measured by flow cytometry. (I-J) On day 6, MDM were stimulated with LPS (10 ng/ml) or irradiated M.tb (10 μg/ml) for 24 hours. (I) Cell viability (relative to untrained unstimulated MDM) was determined using crystal violet staining. (J) The relative expression of *TBET* (compared to untrained) in unstimulated MDM measured by qPCR. (K) The concentration of IFN-γ in the supernatant was measured by ELISA. (L) Cells were left untrained (white) or trained with IFN-γ or IL-17 (1 ng/ml: pink; 10 ng/ml: green; 100 ng/ml: purple). On day 6, cells were stimulated with M.tb for 24 hours. The concentration of IL-1β was measured by ELISA. (N-O) On days 7, 14, 21 and 28, cells were stimulated with M.tb for 24 hours. The concentration of (N) IL-6 and (O) IL-1β was measured by ELISA. Each dot represents an individual donor (A-C, I) n=6, (D, J, K, L) n=3, or (E-F) n=5. Data are graphed as paired data joined by a line or the mean value ± SD. *p<0.05 or ***p<0.001 determined using a (A-H, K) paired t test or (I, J, L) two-way ANOVA with (I, J) Šídák's multiple comparisons test or (L-N) Tukey's multiple comparisons test. (N-O) #p<0.05 denotes a paired t test comparing UT with IFN-γ trained MDM in the absence of other timepoints.

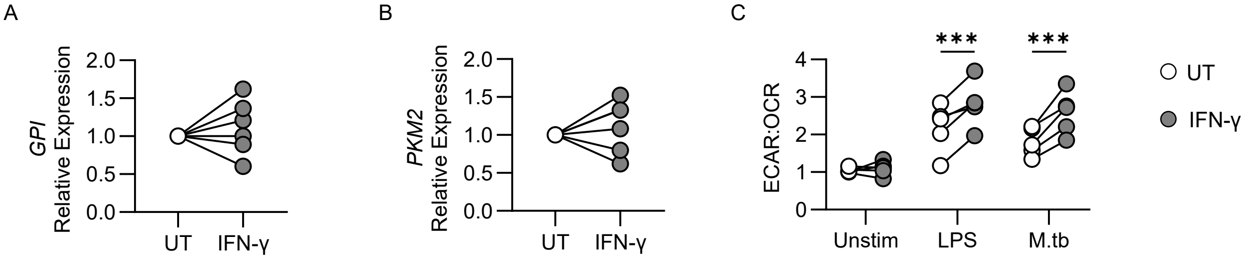

Supplemental Figure 3:
Enriched monocytes were left untrained (UT: white) or were trained with IFN-γ (10 ng/ml: grey) for 24 hours. Cells were then differentiated into MDM. (A-B) Relative expression (compared to untrained) of (A) *GPI*, or (B) *PKM2* in unstimulated MDM on day 7 measured by qPCR. (C) On day 6, MDM metabolism was assessed using the Seahorse XFe24 analyser. Data shows the ratio of the relative (compared to untrained unstimulated) change in ECAR to the relative (compared to untrained unstimulated) change in OCR in IFN-γ trained MDM when stimulated with LPS (10 ng/ml) or irradiated M.tb (10 µg/ml). Each dot represents an individual donor (A) n=6 or (C) n=5 with paired data joined by a line. ***p<0.001 determined using a (A-B) paired t test or (C) two-way ANOVA with Šídák’s multiple comparisons test.

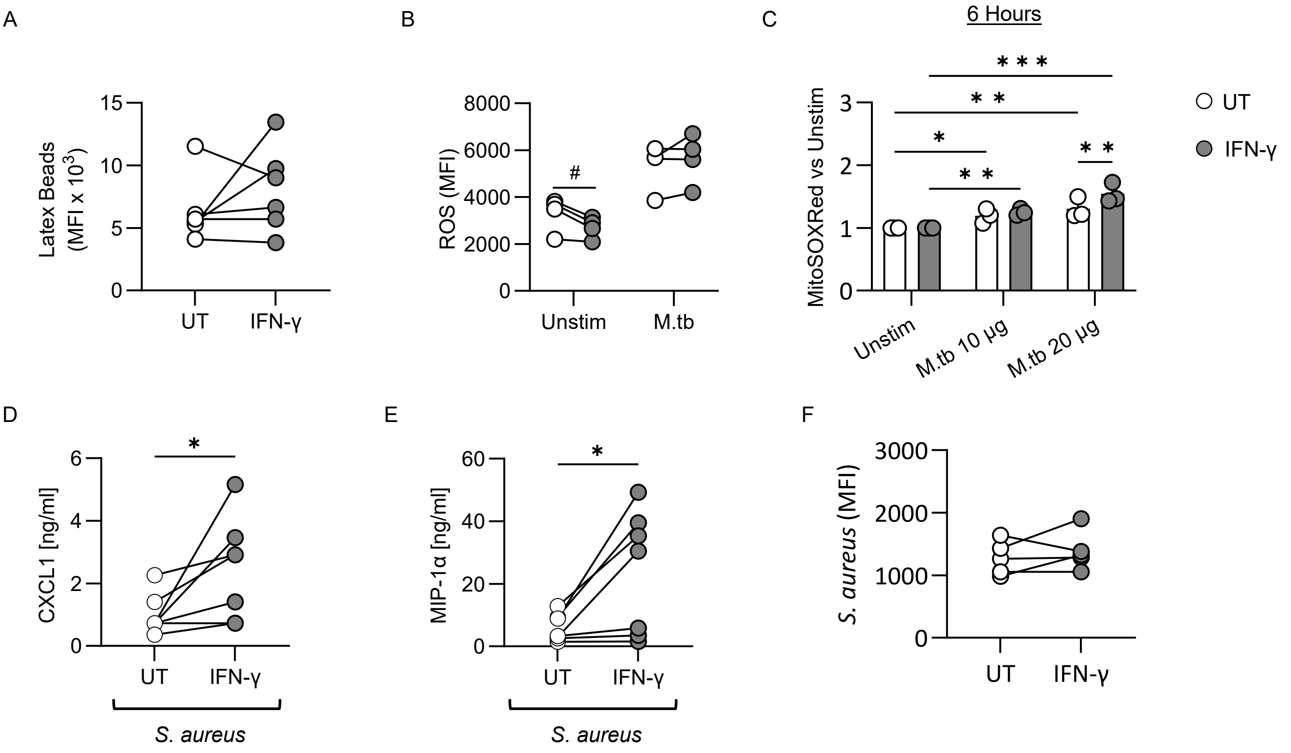

Supplemental Figure 4:
Enriched monocytes were left untrained (UT: white) or were trained with IFN-γ (10 ng/ml: grey) for 24 hours. Cells were then differentiated into MDM. On day 7, MDM were (A) left uninfected, (B-C) stimulated with irradiated M.tb (10-20 µg/ml), or (D-E) infected with *S. aureus* (USA300; MOI 100:1) for the indicted timepoints. (A) MDM phagocytosis was measured using latex bead uptake and assessed by flow cytometry. (B) MDM were stimulated with M.tb and ROS was measured using DHR123 staining and assessed by flow cytometry. (C) MDM were stimulated with M.tb and mitochondrial ROS was measured using MitoSOX staining and assessed by flow cytometry. (D-E) The concentration of (D) CXCL1 or (E) MIP-1α measured by ELISA 24 hours post *S. aureus* infection. (F) The MFI of MDM infected with CFSE labelled *S. aureus* (USA300; MOI 100:1). Each dot represents an individual donor (A) n=6, (B) n=4, (C) n-3, (D-E) n=7, or (F) n=5. (A, D-F) with paired data joined by a line. *p<0.05, **p>0.01, ***p<0.001 determined using a (A-B, D-E) paired t test or (C) two-way ANOVA with uncorrected Fisher's LSD test. (B) Data was analysed by two-way ANOVA, however results were not statistically significant. #p<0.05 denotes a paired t test comparing UT with IFN-γ trained MDM in the absence of M.tb stimulation.

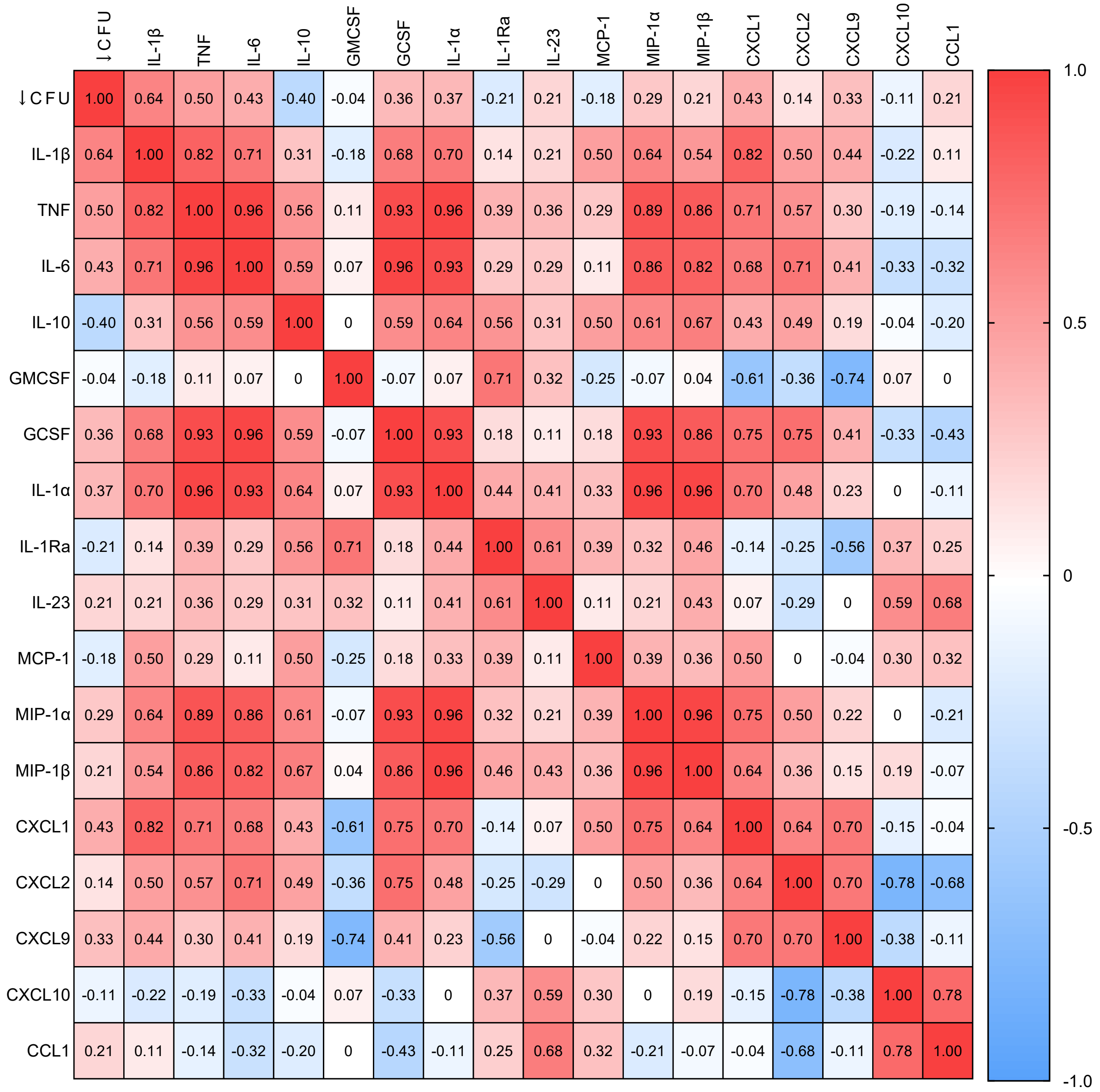

Supplemental Figure 5:
Enriched monocytes were left untrained or were trained with IFN-γ (10 ng/ml) for 24 hours. Cells were then differentiated into MDM. On day 7, MDM were infected with *S. aureus* (USA300; MOI of 100:1) for 24 hours. The concentration of IL-1β, TNF, IL-6, IL-10, GMCSF, GCSF, IL-1α, IL-1Ra, IL-23, IP-10, MCP-1, MIP-1α, MIP-1β, CXCL1, CXCL2, CXCL9 and CCL1 was measured using a custom Luminex Discovery Assay. All data was input as the fold change of each readout in the IFN-γ trained cells. This was calculated by dividing the IFN-γ trained data by the untrained data. Red denotes a positive correlation, and blue denotes a negative correlation. Data is shown as collated data from n=7 matched donors. Spearman correlation analysis was used to generate correlation coefficient values (r values)

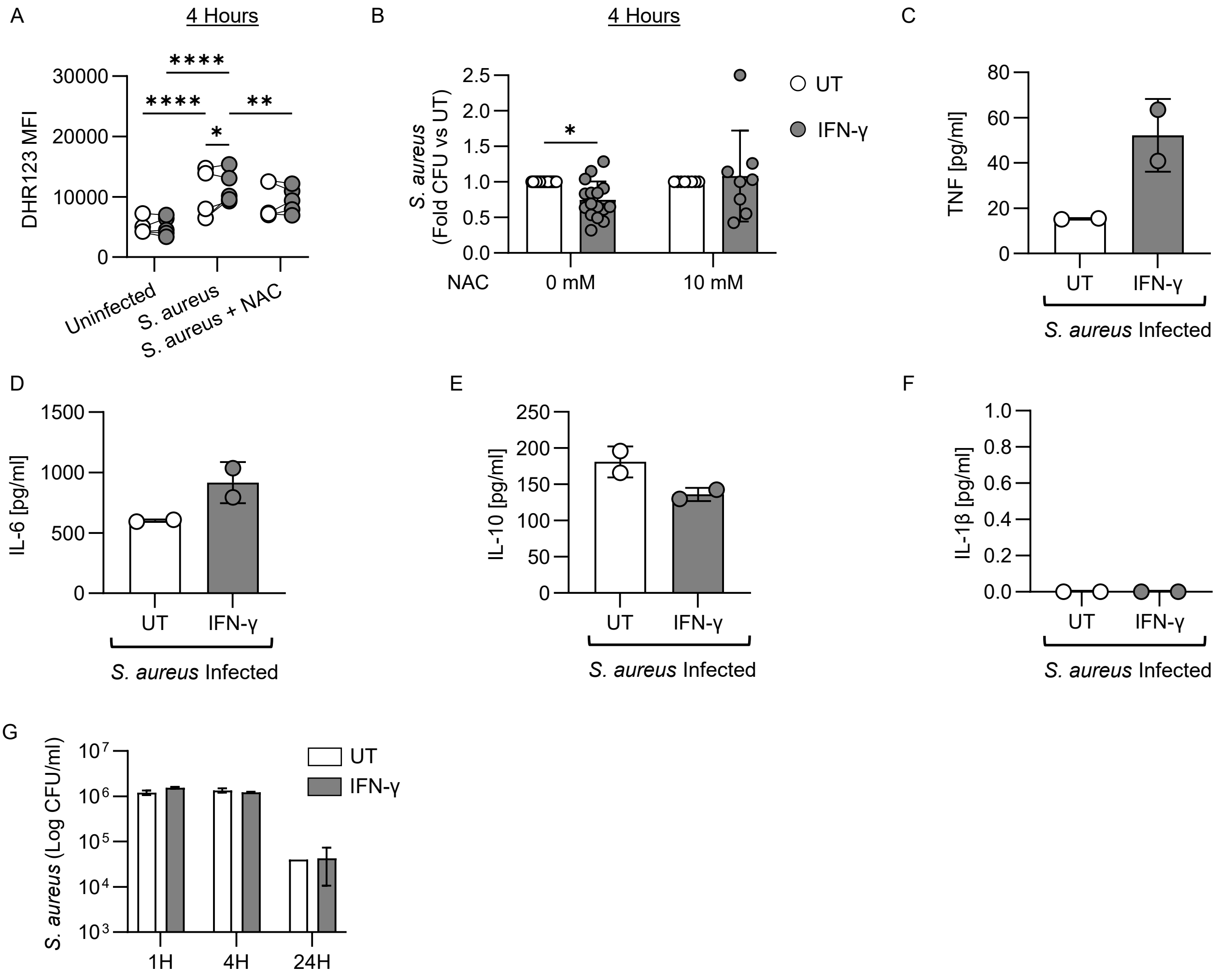

Supplemental Figure 6:

Enriched monocytes were left untrained (UT: white) or were trained with IFN-γ (10 ng/ml: grey) for 24 hours. Cells were then differentiated into MDM. On day 7, MDM were infected with *S. aureus* (USA300; MOI 100:1) for the indicted timepoints. (A) ROS was measured in cells treated with 0 mM or 10 mM NAC using DHR123 staining and assessed by flow cytometry. (B) The fold change in the CFU/ml of S. aureus (USA300; MOI 100:1) compared to the UT dataset in cells treated with 0 mM or 10 mM NAC. (C-F) The concentration of (C) TNF, (D) IL-6, (E) IL-10, or (F) IL-1β was measured by ELISA 24 hours post *S. aureus* infection in MDM from a CGD patient. (G) The bacterial burden of *S. aureus* (USA300; MOI 100:1) within CGD patient MDM was measured by CFU enumeration at 1-, 4-, or 24-hours post-infection. Data are graphed as paired data joined by a line or the mean value ± SD. (A) n=16, or (B-F) n=1. *p<0.05 determined using (A-B) two-way ANOVA with uncorrected Fisher's LSD test.

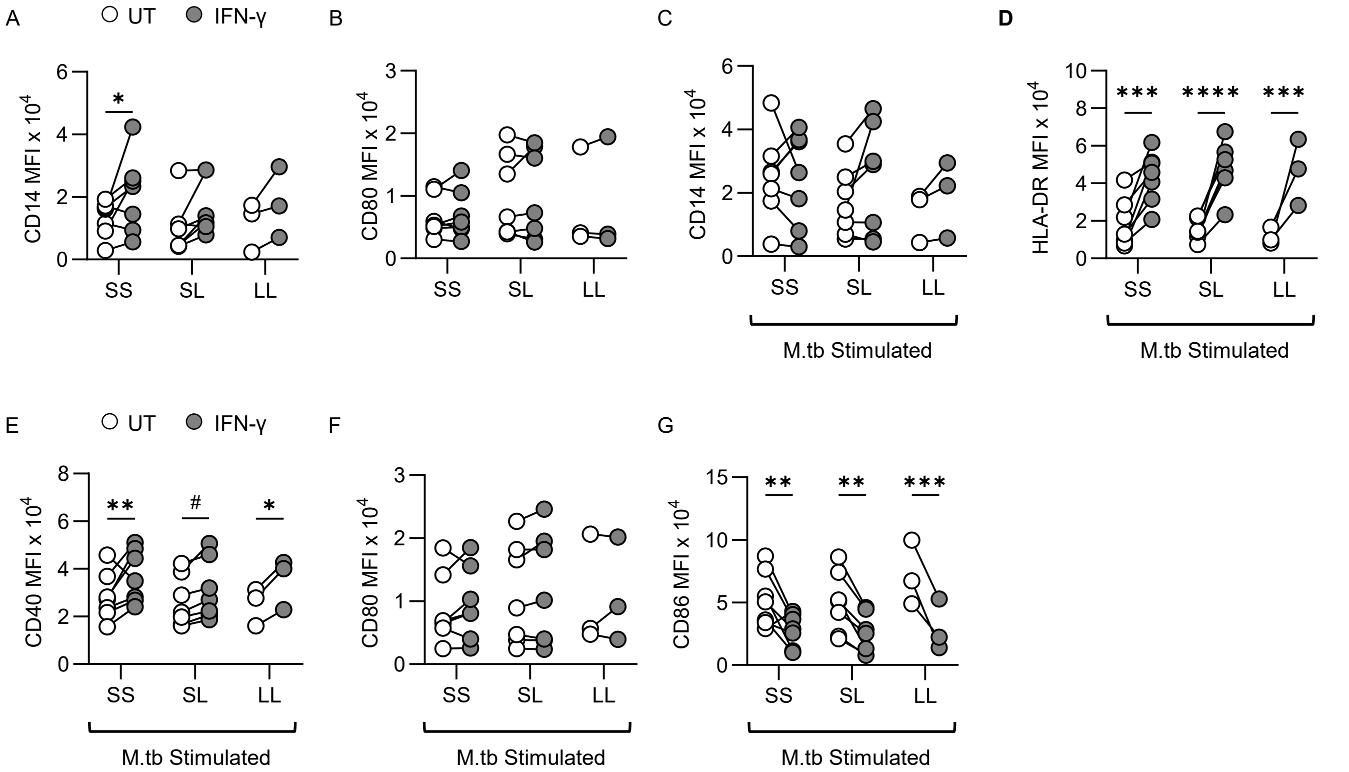

Supplemental Figure 7:
Enriched monocytes from donors with different genotypes of the *TIRAP* S180L polymorphism were left untrained (UT: white) or were trained with IFN-γ (10 ng/ml: grey) for 24 hours. Cells were then differentiated into MDM. (C-G) On day 6, MDM were stimulated with irradiated M.tb (0 μg/ml) for 24 hours. (A-B) The expression of (A) CD14 or (B) CD80 was measured by flow cytometry on unstimulated MDM on day 7 grouped by *TIRAP* S180L genotype. (C-G) The expression of (C) CD14, (D) HLA-DR, (E) CD40, (F) CD80 or (G) CD86 measured by flow cytometry in response to M.tb on day 7 grouped by *TIRAP* S180L genotype. TIRAP S180 homozygous (wildtype; SS, n = 11), S180L heterozygous (SL, n = 8), or S180L homozygous (LL, n = 6) individuals are plotted with each dot representing a single donor and paired data joined by a line. *p<0.05, **p<0.01, ***P<0.001, or ****p<0.0001 determined using a two-way ANOVA with Tukey's multiple comparisons test. (E) No significance measured by two-way ANOVA, #p<0.05 determined using paired t test comparing UT to IFN-γ trained MDM in SL donors alone.

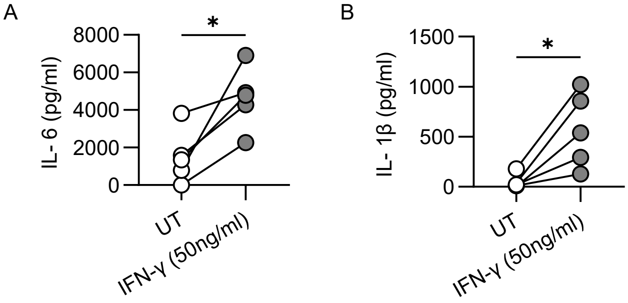

Supplemental Figure 8:

Sorted monocytes were left untrained (UT) or were trained with IFN-γ (IFN; 50 ng/ml) for 24 hours. Cells were then differentiated into MDM. On day 7, cells were stimulated with M.tb for 24 hours. The concentration of (A) IL-6 and (B) IL-1β was measured by ELISA. Each dot represents an individual donor n=5 with paired data joined by a line. *$p<0.05$ determined using paired t test.
