# Supplementary figures and images for: IFN-γ–induced trained immunity enhances killing of priority pathogens in healthy and genetically vulnerable individuals
Source: JCI Insight. 2026 Feb 10;11(6):e195866. doi: 10.1172/jci.insight.195866 (PMC13043095; doi:10.1172/jci.insight.195866)

Figure 2A

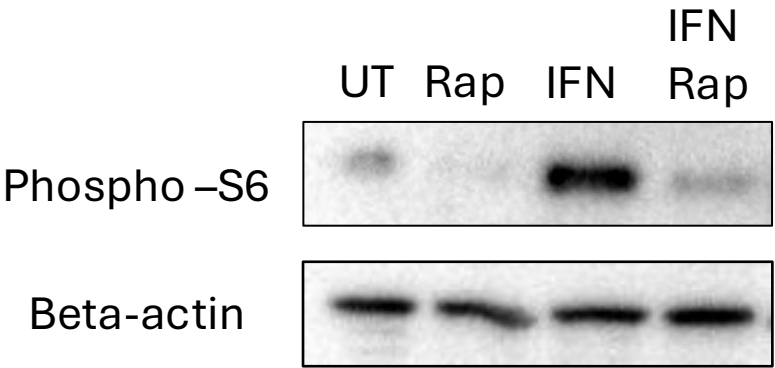

## Donor A

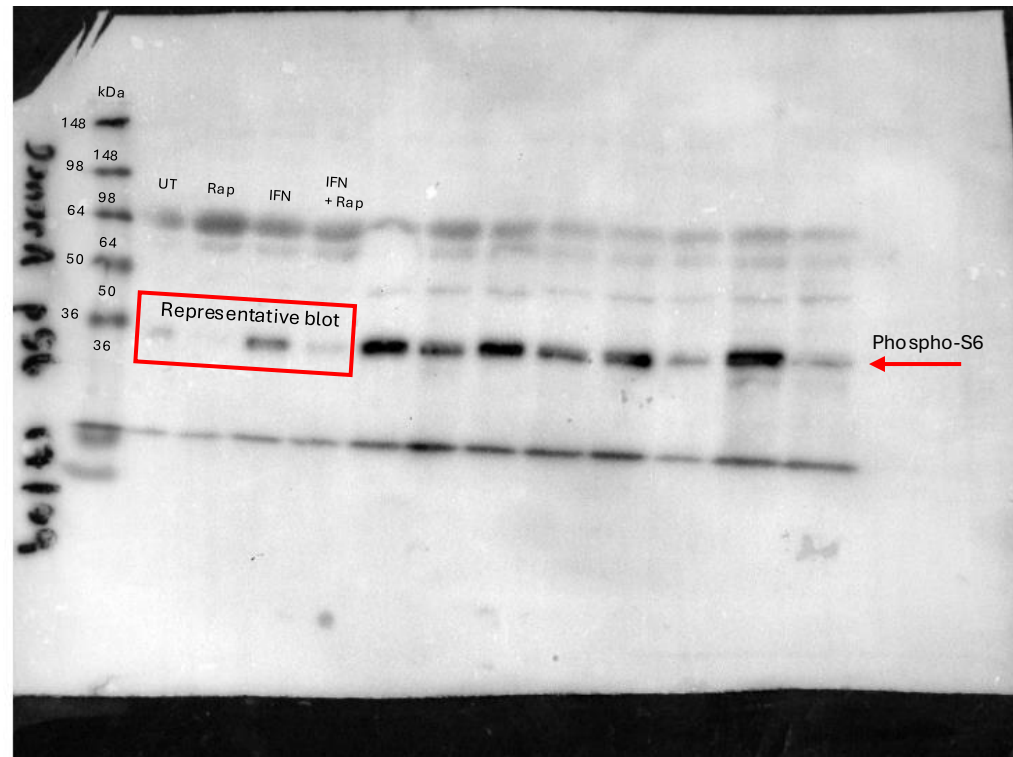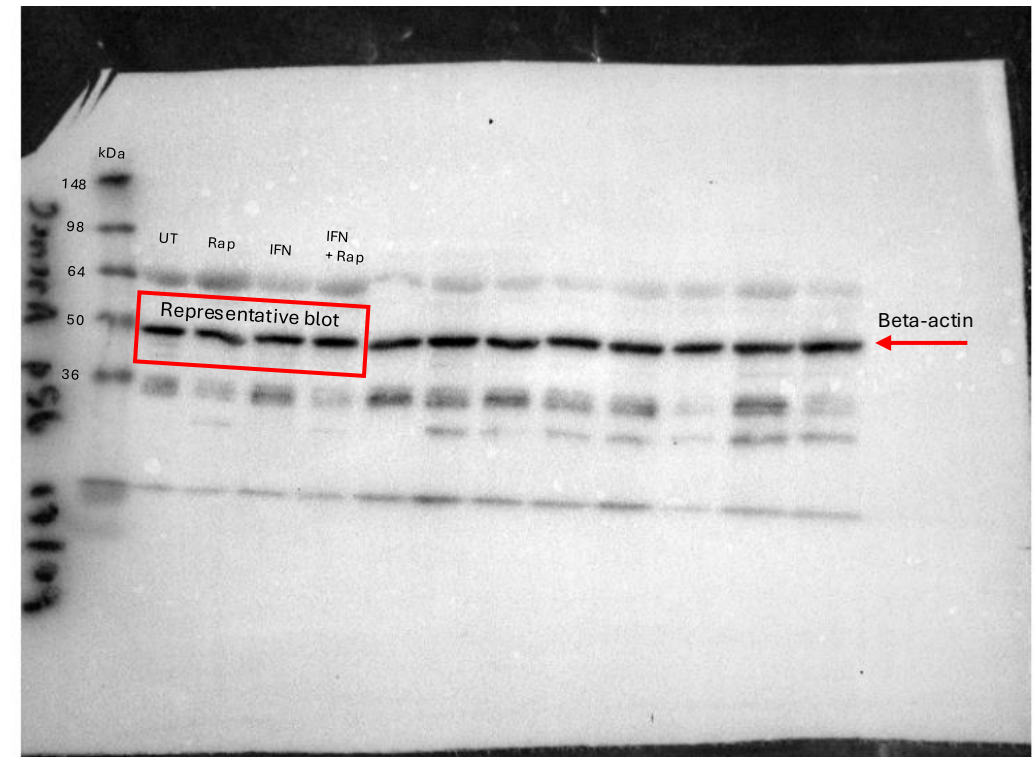

Donor B

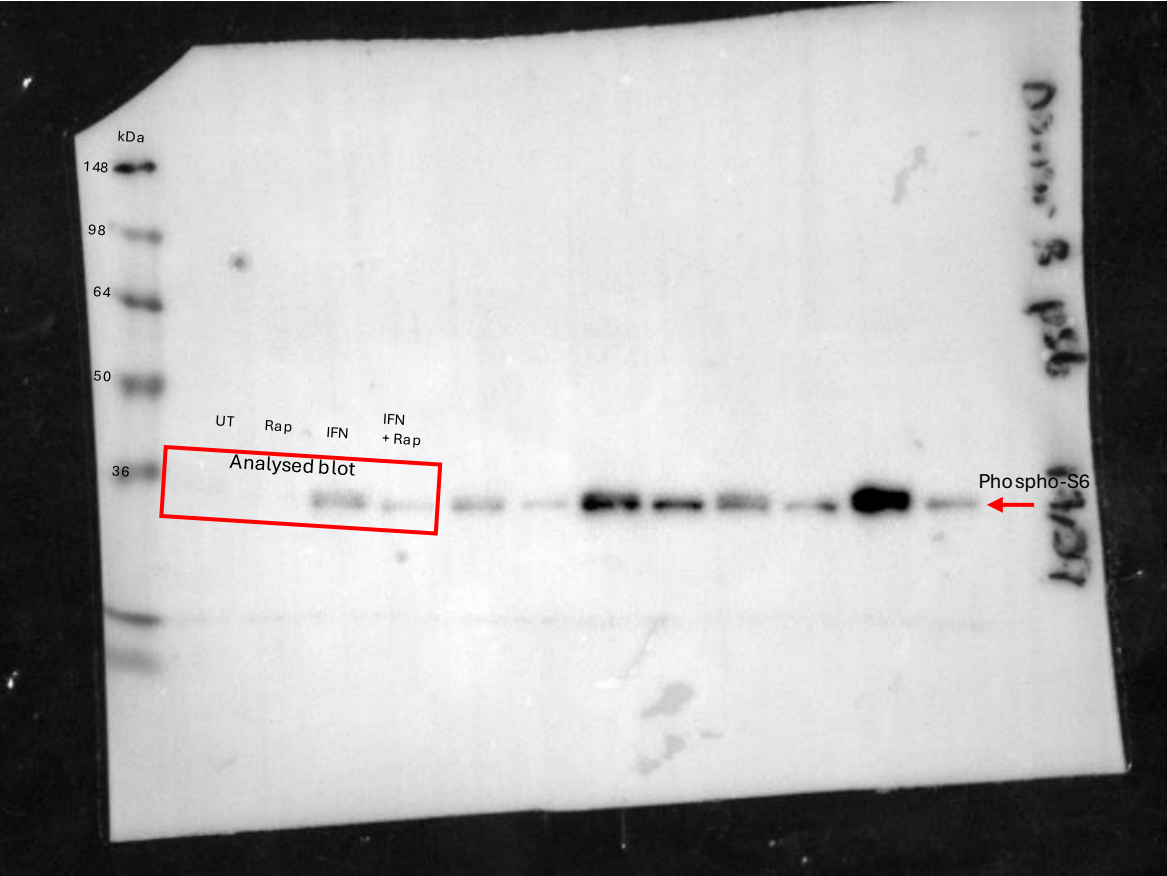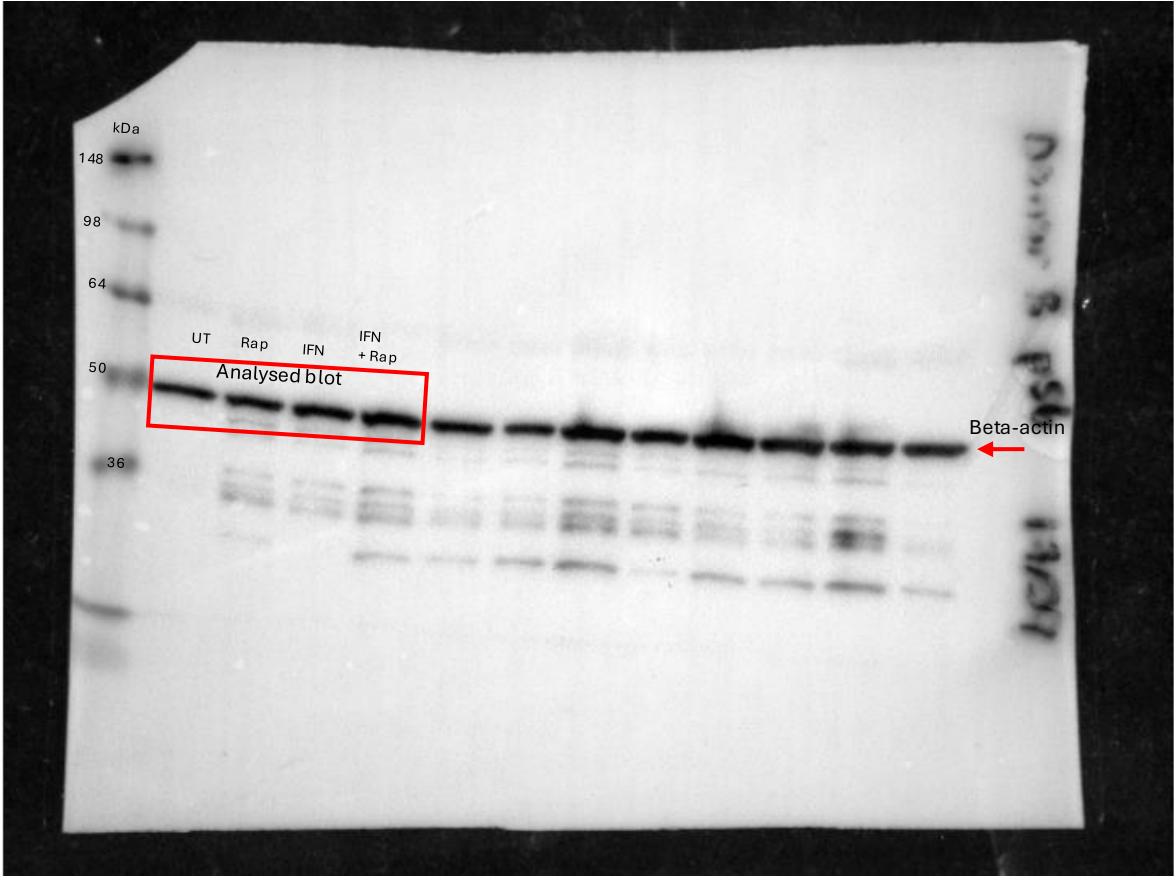

Donor C

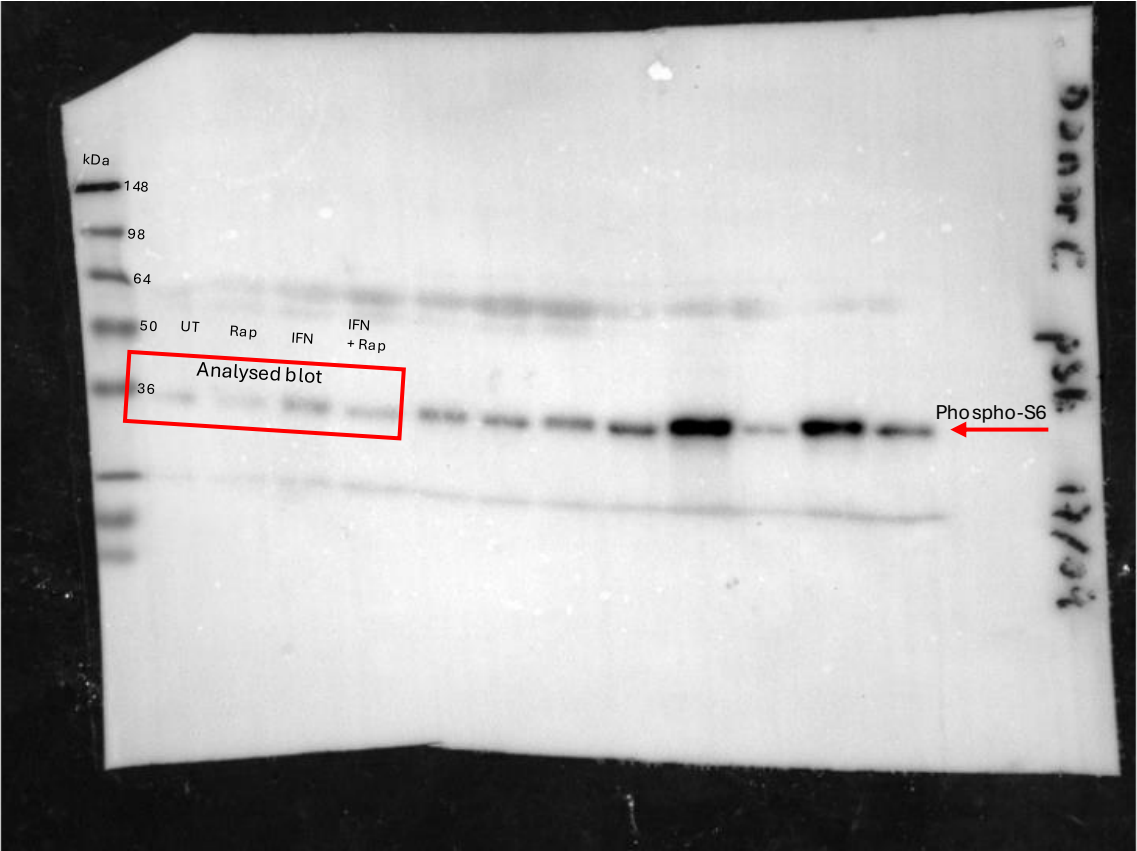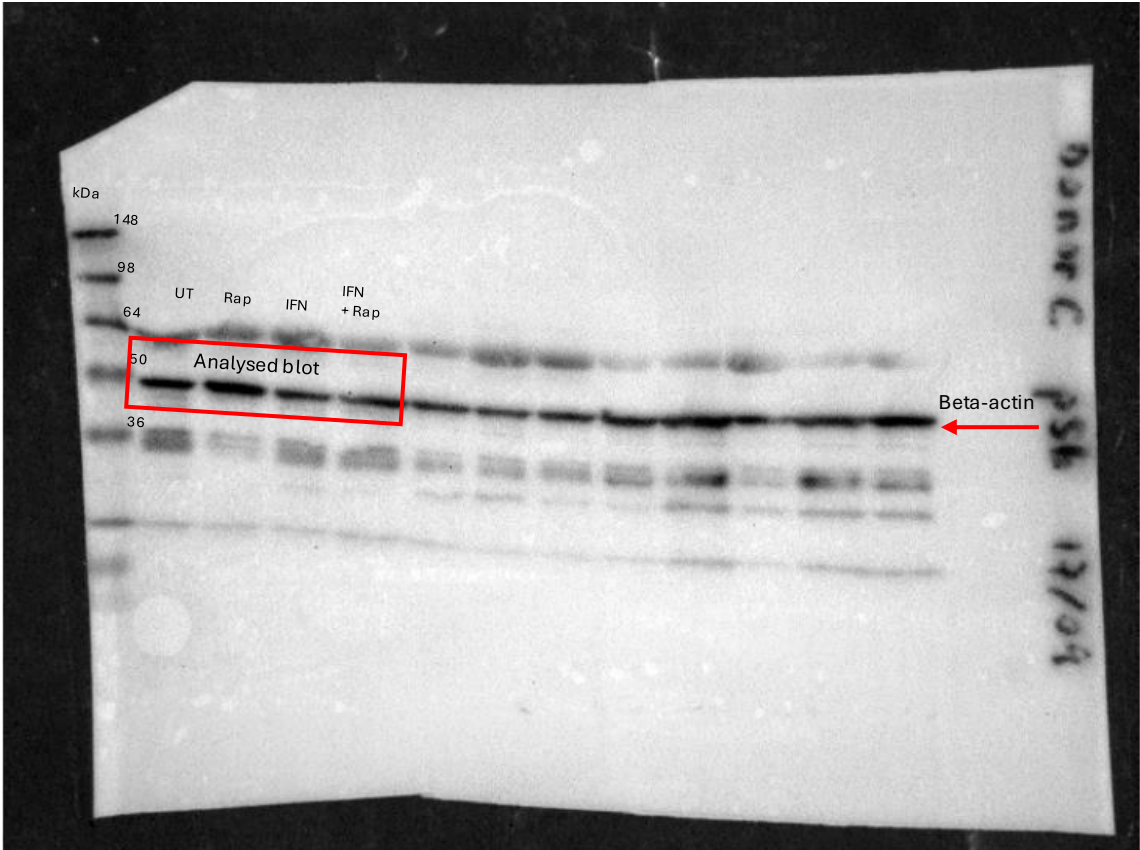

Donor D

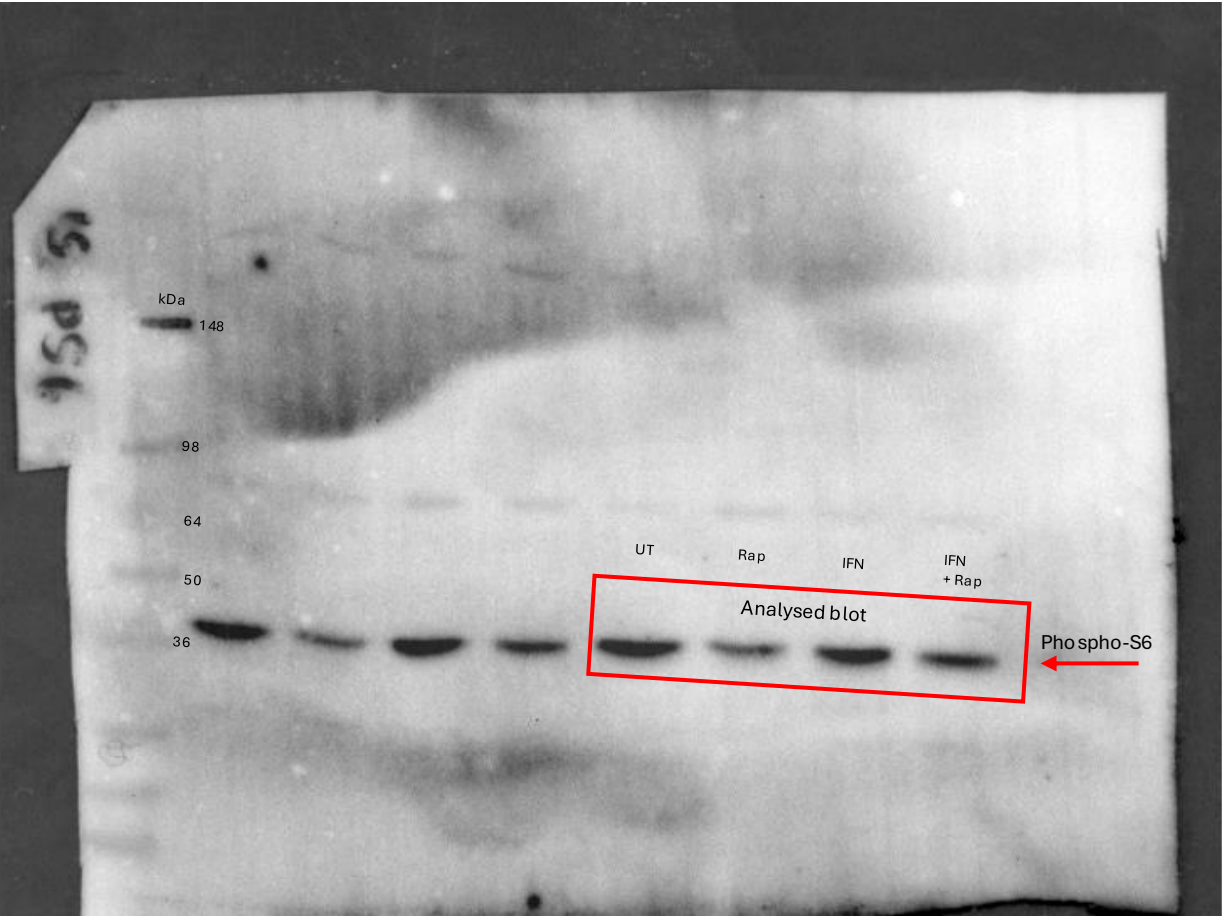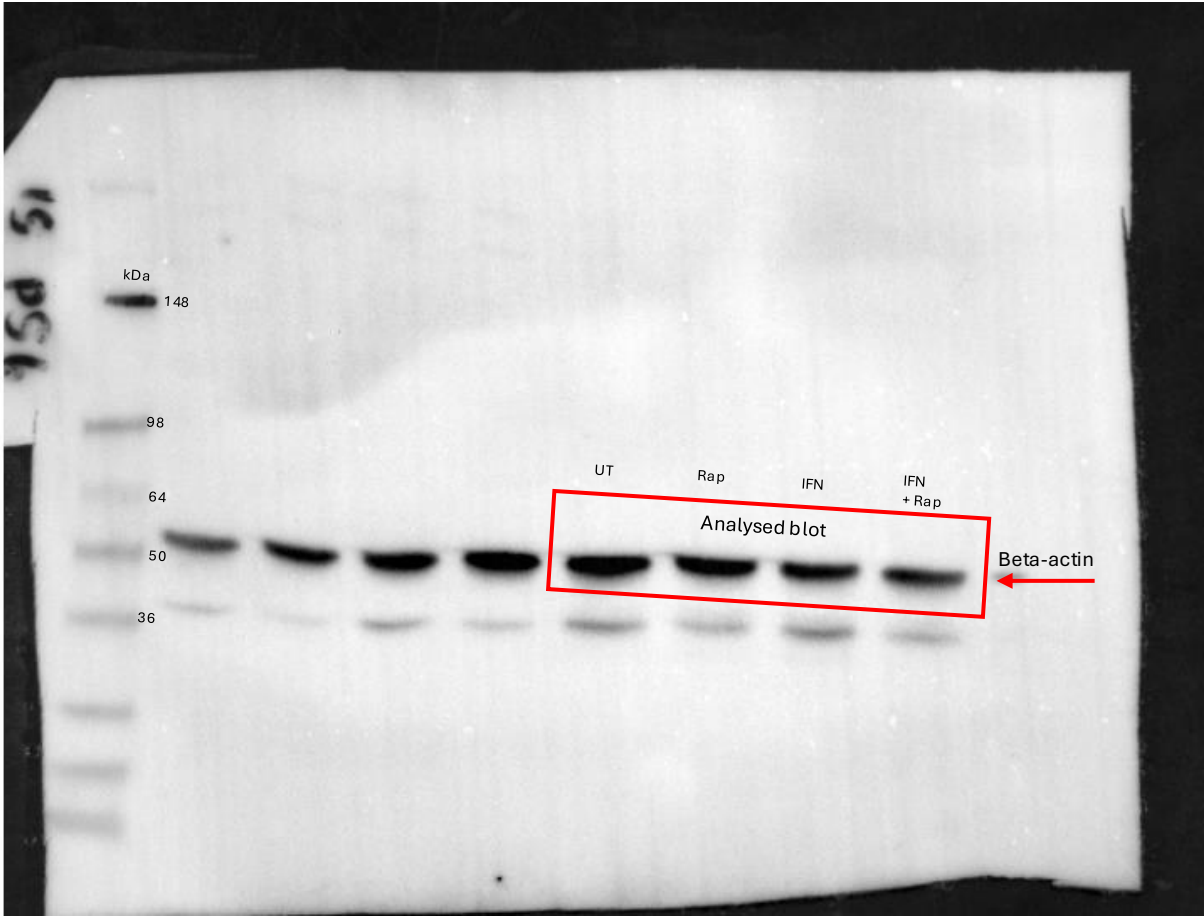

Supplement: Unedited blot and gel images [file jciinsight-11-195866-s065.pdf]
